# *Sanguisorba officinalis L*. derived from herbal medicine prevents intestinal inflammation by inducing autophagy in macrophages

**DOI:** 10.1038/s41598-020-65306-4

**Published:** 2020-06-19

**Authors:** Asuka Yasueda, Hisako Kayama, Michiko Murohashi, Junichi Nishimura, Koji Wakame, Ken-ichi Komatsu, Takayuki Ogino, Norikatsu Miyoshi, Hidekazu Takahashi, Mamoru Uemura, Chu Matsuda, Toru Kitagawa, Kiyoshi Takeda, Toshinori Ito, Yuichiro Doki, Hidetoshi Eguchi, Shigeomi Shimizu, Tsunekazu Mizushima

**Affiliations:** 1 Department of Gastroenterological Surgery, Graduate School of Medicine, Osaka University Graduate School of Medicine, 2-2 Yamadaoka, Suita, Osaka 565-0871 Japan; 2 Laboratory of Immune Regulation, Department of Microbiology and Immunology, Osaka University Graduate School of Medicine, 2-2 Yamadaoka, Suita, Osaka 565-0871 Japan; 3 Institute for Advanced Co-Creation Studies, Osaka University, 2-2 Yamadaoka, Suita, Osaka 565-0871 Japan; 4 Department of Pathological Cell Biology, Medical Research Institute, Tokyo Medical and Dental University, 1-5-45, Yushima, Bunkyo-ku, Tokyo 113-8510 Japan; 5 Department pharmacology, Hokkaido University of Science, 15-4-1, Maeda shichi-jyo, Teine, Sapporo City, Hokkaido 006-8590 Japan; 6 Integrated Frontier Research for Medical Science Division, Institute for Open and Transdisciplinary Research Initiatives, Osaka University, 1-1 Yamadaoka, Suita, Osaka 565-0871 Japan; 7 Osaka Center for Cancer and Cardiovascular Disease Prevention, 1-6-107, Morinomiya, Jyoto-ku, Osaka City, Osaka, 536-0025 Japan

**Keywords:** Immunology, Diseases, Gastroenterology

## Abstract

Disturbed activation of autophagy is implicated in the pathogenesis of inflammatory bowel disease. Accordingly, several autophagy-related genes have been identified as Crohn’s disease susceptibility genes. We screened the autophagy activators from a library including 3,922 natural extracts using a high-throughput assay system. The extracts identified as autophagy activators were administered to mice with 2% dextran sodium sulfate (DSS). Among the autophagy inducers, *Sanguisorba officinalis L*. (SO) suppressed DSS-induced colitis. To identify the mechanism by which SO ameliorates colitis, epithelial cell and innate myeloid cells-specific *Atg7*-deficient mice (*Villin-cre; Atg7*^*f/f*^ and *LysM-cre; Atg7*^*f/f*^ mice, respectively) were analyzed. SO-mediated inhibition of colitis was observed in *Villin-cre*; *Atg7*^*f/f*^ mice. However, SO and a mixture of its components including catechin acid, ellagic acid, gallic acid, and ziyuglycoside II (Mix_4_) did not suppressed colitis in *LysM-cre*; *Atg7*^*f/f*^ mice. In large intestinal macrophages (Mφ) of *Atg7*^*f/f*^ mice, SO and Mix_4_ upregulated the expression of marker genes of anti-inflammatory Mφ including *Arg1*, *Cd206*, and *Relma*. However, these alterations were not induced in *LysM-cre*; *Atg7*^*f/f*^ mice. These findings indicate that SO and its active components ameliorate DSS-induced colitis by providing intestinal Mφ with anti-inflammatory profiles via promotion of Atg7-dependent autophagy.

## Introduction

Inflammatory bowel diseases (IBD), including Crohn’s disease (CD) and ulcerative colitis (UC), are chronic relapsing-remitting disorders of the gastrointestinal tract. The numbers of patients with CD and UC in Japan were 70,700 and 219,685, respectively, and still increasing according to an analysis of a nationwide survey^1^. IBD patients are on chronic medication, such as 5-aminosalicylates, corticosteroids, and biopharmaceuticals including anti-tumor necrosis factor (TNF) and anti-interleukin (IL)-12/23p40 antibodies^2^. Therapeutic reagents, such as biological drugs and corticosteroids provide therapeutic benefit in the acute inflammation phase, and dramatically suppressed active inflammation. However, in the chronic phase, corticosteroids are ineffective for maintaining remission status. Therefore, development of a cure for IBD that is safe and tolerable in the long-term is needed.

Recent studies have demonstrated that serval herbal medicines are beneficial for suppressing intestinal inflammation. A murine study has reported that indigo naturalis, a traditional herbal formulation, ameliorates intestinal inflammation by promoting production of IL-10 and IL-22 through activation of aryl hydrocarbon receptor^3^. In addition, its anti-inflammatory effect was exerted in human intestine^4^. Among herbal medicines, *Sanguisorba officinalis L*. (SO) has been reported to have several effects, such as anti-oxidative, anti-inflammatory, anti-tumor, anti-allergic, and anti-infection^5–9^. So far, 129 components of SO were identified^8^. Among them, tannins including catechin acid (CA)^10^, ellagic acid (EA)^11^, and gallic acid (GA)^12^ and saponins including ziyuglycoside II (ZY)^13^ have been identified as major active components of SO with anti-inflammatory capacities.

Autophagy plays an important role in the degradation of unnecessary and/or dysfunctional components in lysosome. Microtubule-associated protein light chain 3 (LC3), a mammalian homolog of fungal Atg8, is commonly used to survey autophagy state^14^. LC3-I is converted to LC3-II that is essential for initiation of autophagy. Autophagy related 7 (Atg7), an E1-like enzyme, is an essential molecule for progression of autophagy, because it activates LC3 in an ATP-dependent manner during the autophagosome formation^15^. Recent studies have shown that adequate activation of autophagy prevents intestinal inflammation associated with IBD^16,17^. Furthermore, dysregulated activation of autophagy is implicated in the development and/or pathogenesis of autoimmune disorders, including systemic lupus erythematosus and rheumatoid arthritis^18–20^. Autophagy-related genes, such as *Atg16L1* and *NOD2*, have been reported to be associated with the pathogenesis of CD^16,21,22^. Moreover, *LRRK2* has been identified as a CD-susceptibility gene, especially in Japanese cohorts^23^. These genes are essential for appropriate production of cytokines in macrophages (Mφ)^24,25^. In addition, autophagy contributes to the maintenance of intestinal epithelial stem cells, epithelial regeneration during inflammation^26^, and elimination of bacteria invasion by facilitating intestinal epithelial cells integrity^27^. Recent studies have demonstrated that autophagy activation by rapamycin^25^, trehalose^25^, curucumin^28–31^, or celastol^32^, ameliorates colitis. In addition, several dietary compounds and herbal medicines activate autophagy and thereby inhibiting intestinal inflammation and maintain intestinal homeostasis^25^. Thus, precise analysis about autophagy regulator might link to development of novel therapeutic interventions in IBD.

Because Mφ and dendritic cells (DCs) instruct the adaptive immune system by producing pro- and anti-inflammatory cytokines, disrupted innate immune responses lead to inadequate activation of helper T (Th) cells, such as IFN-γ-producing CD4^+^ (Th1) cells and IL-17-producing CD4^+^ (Th17) cells. Accordingly, IBD patients show dysregulated Th1 and Th17 cell responses accompanied with increased number of inflammatory Mφ and DCs with promoted antigen presentation and enhanced production of pro-inflammatory cytokines in the intestinal mucosa^33,34^. In the steady state, anti-inflammatory CX_3_CR1^high^ Mφ are abundantly present in murine intestine, whereas inflammatory DCs including CX_3_CR1^intermediate^ DCs increase under the inflammatory condition^35,36^. To maintain intestinal mucosal tolerance, development and genes expression of Mφ and DCs are tightly regulated by various mechanisms in the intestine^35,36^. For instance, anti-inflammatory cytokine IL-10 produced by Mφ and Foxp3^+^ regulatory T (Treg) cells negatively regulates expression of pro-inflammatory cytokines including *Il23a*, *Il6*, *Il12b*, *Tnf* in intestinal Mφ and DCs^37–39^.

In the present study, we screened for potential autophagy activators contributing to the prevention of intestinal inflammation. As a result, we identified that SO inhibits intestinal inflammation through facilitating Atg7-dependent Mφ autophagy.

## Results

### Administration of SO ameliorates dextran sodium sulfate (DSS)-induced colitis

**Figure 1:**
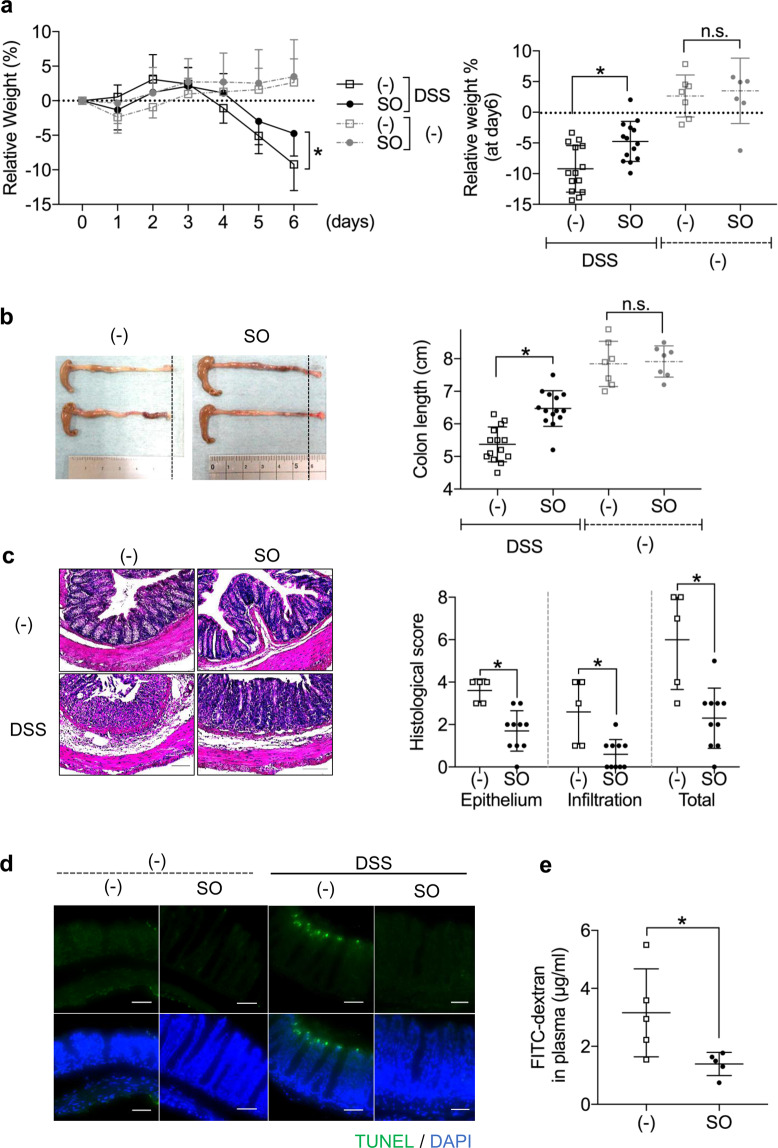
An autophagy activator SO ameliorated DSS-induced colitis. C57BL/6J mice were administered either SO or vehicle with (n = 14) or without (n = 7) 2% DSS for 6 days. (**a**) Body weight changes (means ± SD). (**b**) Colon length 7 days after starting DSS administration (means ± SD). (**c**) Representative histopathological images of distal colons (left) and histopathological score (right) (means ± SD from three independent experiments). Data were analyzed by Dunnet’s test, *p < 0.05. Bar; 100 μm. (**d**) Representative images of TUNEL staining of the colon 6 days after DSS administration. All data are representative of three independent experiments. (**e**) Concentration of FITC-dextran in the plasma (n = 5 in each group).

To identify autophagy activator that control intestinal inflammation, we first performed a high-throughput assay (Supplementary Fig. S1). Among 3922 natural extracts including several herbal medicines, we identified 33 as inducers of autophagy. To determine effects of these products on the regulation of intestinal inflammation, mice were administered DSS in the presence or absence of these autophagy activators. Among them, *Sanguisorba officinalis L*. (SO) at a dose of 1 mg/ml, but not 0.1 and 0.01 mg/ml, markedly suppressed weight loss and colon shortening during DSS-induced colitis without tissue damage in the liver, heart, and kidney (Supplementary Fig. S2a–c): hereafter, we administered 1 mg/ml of SO to mice. Seven days after starting DSS administration, the severe weight loss and colon shortening were inhibited by co-administration of SO (Fig. 1a,b), which was associated with less colonic histopathology including mild inflammatory cell infiltration (Fig. 1c). Terminal deoxynucleotidyl transferase dUTP nick end labeling (TUNEL) staining showed that the epithelial cell apoptosis observed in mice administered DSS was greatly suppressed in the presence of SO (Fig. 1d). In this context, SO administration led to significantly less translocation of FITC-dextran into the blood (Fig. 1e). Posttreatment with SO led to promotion of tissue recovery in mice suffered from DSS-induced colitis. (Supplementary Fig. S3a). However, pretreatment with SO did not affect initiation of inflammation and tissue repair in the intestine (Supplementary Fig. S3b). These results demonstrate that co-administration of SO with DSS lead to inhibition of intestinal inflammation.

### Activation of Atg7-dependent epithelial autophagy by SO linked to partial suppression of DSS-induced colitis

**Figure 2:**
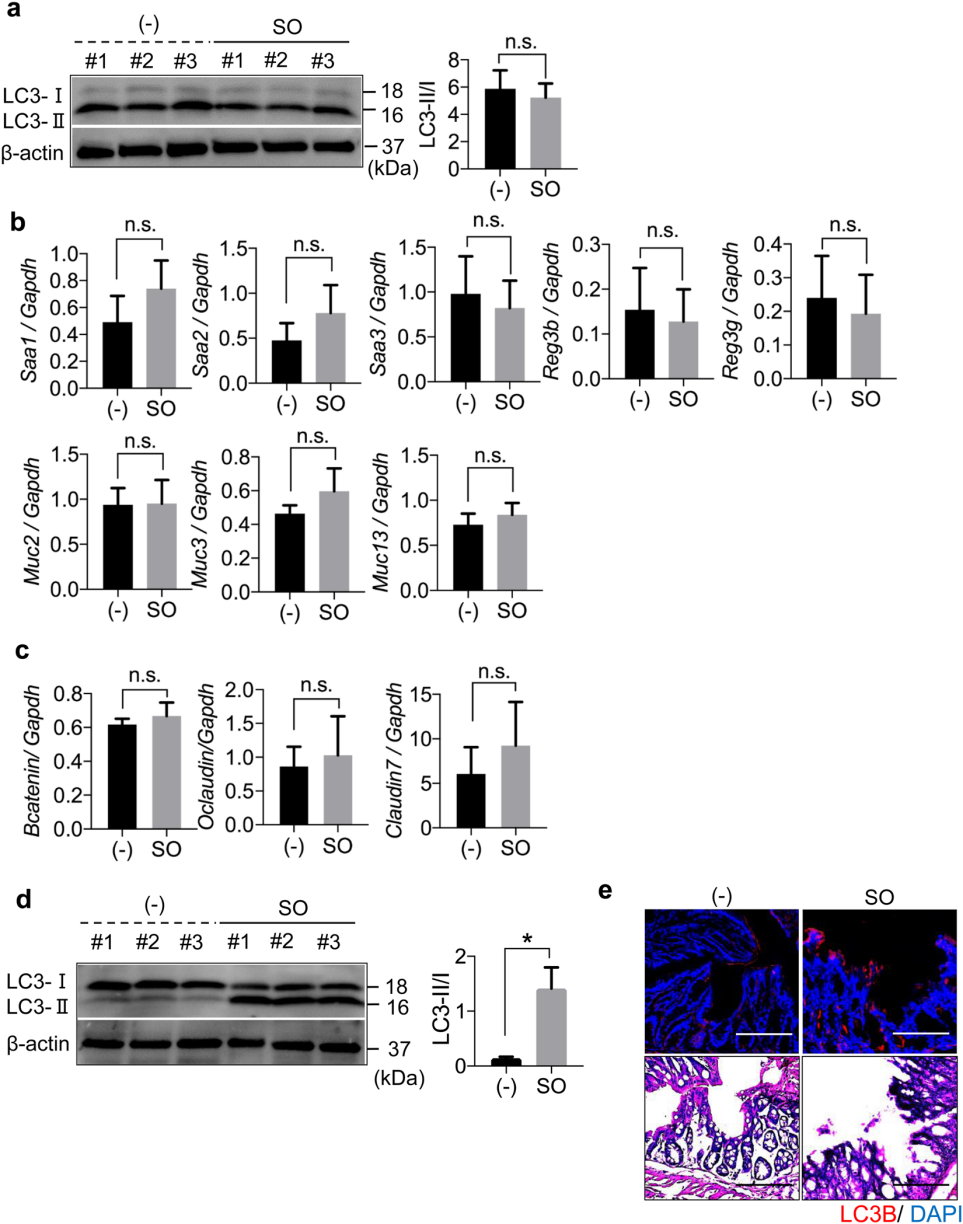
SO did not induce autophagy in large intestinal epithelial cells in the steady state. (**a**) Activation of autophagy by SO in colonic epithelial cells was analyzed by measuring the ratio of LC3-II to LC3-I that detected by western blot analysis using the indicated antibodies. Conversion of LC3 in colonic epithelial cells from C57BL/6J mice administered SO in drinking water for 7 days. The ratio of LC3-II to LC3-I was measured with Image studio lite ver. 5.2 (Li-Cor Bioscience, Nebraska, USA). Full-length blots are shown in Supplementary Fig. S10a. (**b**,**c**) Expression levels of the indicated genes in large intestinal epithelial cells from mice treated or untreated with SO in the steady state. Data are mean ± SD from three independent experiments. Data were analyzed by unpaired t-test. n.s., not significant. (**d**) Conversion of LC3 in colonic epithelial cells from C57BL/6J mice administered DSS with or without SO in drinking water for 7 days. *P < 0.05. Full-length blots are shown in Supplementary Fig. S10b. All data are representative of three independent experiments. (**e**) Expression of LC3B in the colon of C57BL/6J mice administered 2% DSS with or without SO in drinking water for 7 days were analyzed with immunohistochemistry using anti-LC3B antibody (upper). Representative images of H&E staining (bottom). Data are representative of three independent experiments. Bars; 100 μm.

**Figure 3:**
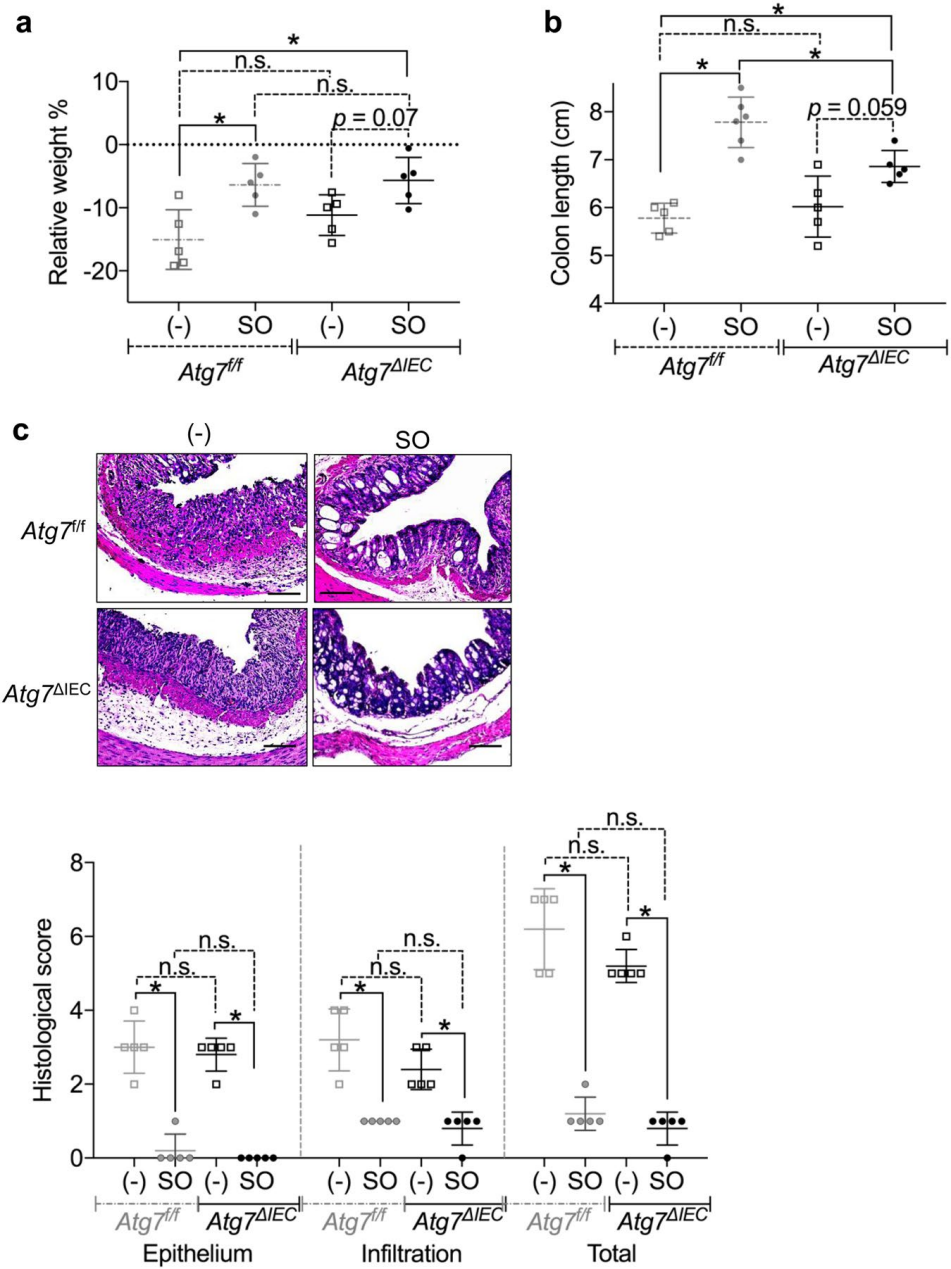
SO-induced autophagy in epithelial cells did not affect intestinal inflammation. *Atg7*^*f/f*^ and *Atg7*^ΔIEC^ mice were administered 2% DSS with or without SO in drinking water for 7 days. (**a**–**c**) Weight changes (**a**), colon length (**b**), (**c**) representative colon sections (upper), and histological score of the colon (bottom) (mean values ± SD). Data are of two independent experiments. Data were analyzed by un-paired t-test. n = 5 in each group. *p < 0.05. n.s, not significant. Bars; 100 μm.

Previous studies have demonstrated that autophagy is involved in intestinal epithelial homeostasis^40,41^. Therefore, we analyzed whether SO-induced autophagy controls epithelial barrier integrity. To examine whether SO-mediated autophagy is occurred in intestinal epithelial cells in the steady state, we administered SO to mice and analyzed induction of epithelial autophagy. SO administration did not alter the ratio of LC3-II to LC3-I in large intestinal epithelial cells, indicating that epithelial autophagy is not enhanced in mice in the steady state condition (Fig. 2a). In accordance, the expression of genes encoding anti-microbial peptides, mucins, and epithelial tight junction-related molecules was not changed by SO administration in the steady state (Fig. 2b,c). In contrast, co-administering of SO with DSS led to markedly increased ratio of LC3-II to LC3-I and promoted expression of LC3B in epithelial cells of the colon (Fig. 2d,e). To elucidate whether SO-mediated activations of epithelial autophagy during DSS administration is responsible for preventing intestinal inflammation, epithelial cell-specific *Atg7* deficient mice (*villin-cre*; *Atg7*^*f/f*^: hereafter called *Atg7*^ΔIEC^ mice) were analyzed (Fig. 3a–c). Similar to *Atg7*^*f/f*^ mice, *Atg7*^ΔIEC^ mice suffered from severe weight loss with shortened colon length, which was associated with worsened histopathology including increased removal of epithelial cells and infiltration of inflammatory cells following DSS administration. SO administration decreased all of clinical parameters with a remarkable resolution of intestinal pathology in *Atg7*^*f/f*^ mice. In *Atg7*^ΔIEC^ mice, SO treatment linked to reduced intestinal histopathology and modest improvement of weight loss and colon shortening. These findings demonstrate that activation of Atg7-dependent epithelia autophagy is partially implicated in SO-mediated suppression of intestinal inflammation.

### SO activates autophagy in intestinal Mφ during DSS-induced colitis

**Figure 4:**
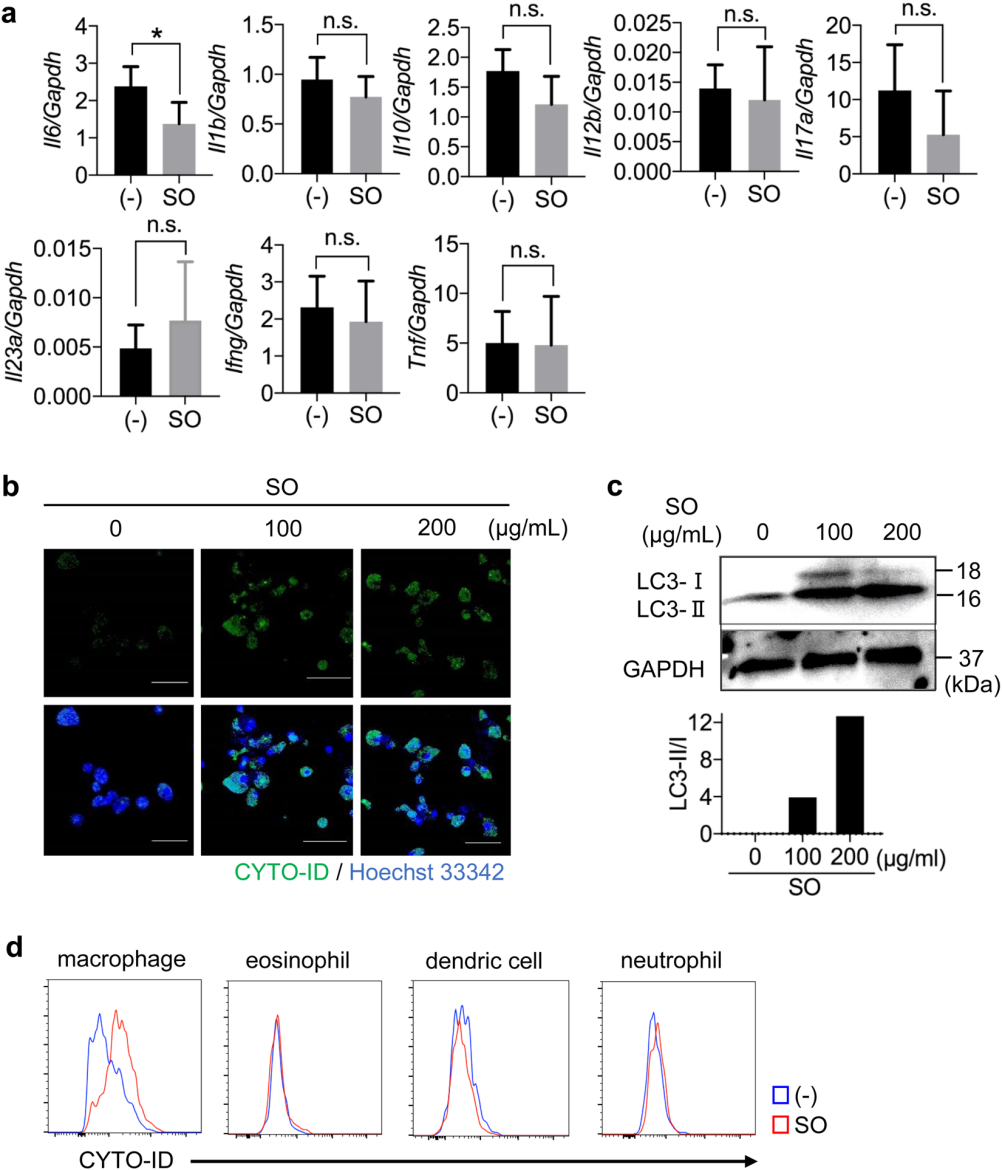
SO induced autophagy in bone marrow-derived Mφ (BMMφ) and intestinal Mφ. (**a**) Expression of the indicated genes in colonic lamina propria cells from mice treated with or without SO in the steady state. Data are mean ± SD of three independent experiments. Data were analyzed by unpaired t-test, *p < 0.05. n.s., not significant. (**b**) CYTO-ID staining in BMMφ treated with or without SO for 24 hours. (**c**) BMMφ were cultured in the presence or absence of SO for 24 hours. The immunoblotting was performed with the indicated antibodies. The graphs show the ratio of LC3-II to LC3-I. Full-length blots are shown in Supplementary Fig. S10c. (**d**) C57BL/6J mice administered 2% DSS with or without SO in drinking water for 7 days and colonic lamina propria cells were isolated. The autophagosome formation stained by CYTO-ID in macrophages, eosinophils, dendric cells and neutrophils were analyzed with flow cytometry. Data are representative of two independent experiments.

Under the steady state conditions, SO administration led to reduced expression of *Il6*, but not *Il1b*, *Il10*, *Il12b*, *Il17a*, *Il23a*, *Ifng* and *Tnf*, in cells from the colonic lamina propria (Fig. 4a). Several studies have demonstrated that autophagy in Mφ is essential for controlling inflammatory responses^24,25,42^. In the intestine, Mφ is a major source of IL-6^43,44^. To define whether SO induces autophagy in Mφ, bone marrow derived Mφ (BMMφ) were cultured in the presence or absence of SO and stained with CYTO-ID, which labels autophagic compartments. CYTO-ID-labeled BMMφ were observed after SO treatment (Fig. 4b). In accordance, the ratio of LC3-II to LC3-I was increased in BMMφ treated with SO in a dose-dependent manner (Fig. 4c), indicating that SO activates autophagy in BMMφ. We further analyzed SO-dependent autophagy in several subsets of innate myeloid cells of the colon. SO administration resulted in increased CYTO-ID-labeled cells in a subset of CD11b^+^ CX_3_CR1^high^ Mφ, but not neutrophils, eosinophils, and CD11b^+^ DCs, from the colon of mice administered DSS (Fig. 4d and Supplementary Fig. S4a–d). These results demonstrate that SO promotes autophagy in large intestinal Mφ.

### SO prevents intestinal inflammation by regulating autophagy in intestinal Mφ

**Figure 5:**
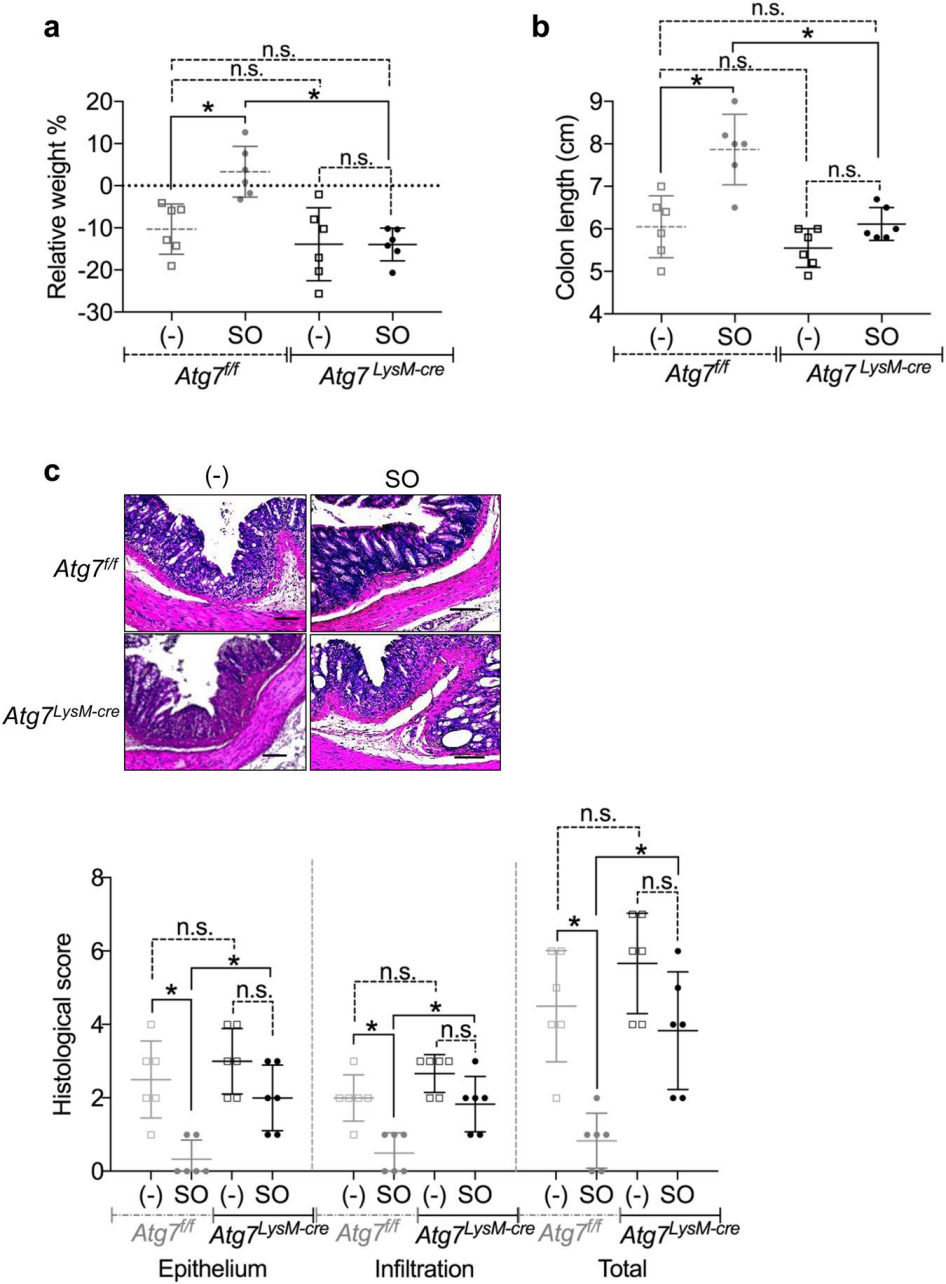
SO did not prevent intestinal inflammation in *Atg7*^*LysM-cre*^ mice. *Atg7*^*f/f*^ and *Atg7*^*LysM-cre*^ mice were administered 2% DSS with or without SO for 7 days. **(a**–**c)** Weight changes (**a**), colon length (**b**), representative colon sections (upper), and histological score of the colon (bottom) (**c**) (mean values ± SD). *p < 0.05. n.s, not significant. n = 6 in each group. Bars; 100 μm. Data are of two independent experiments. Data were analyzed by un-paired t-test.

**Figure 6:**
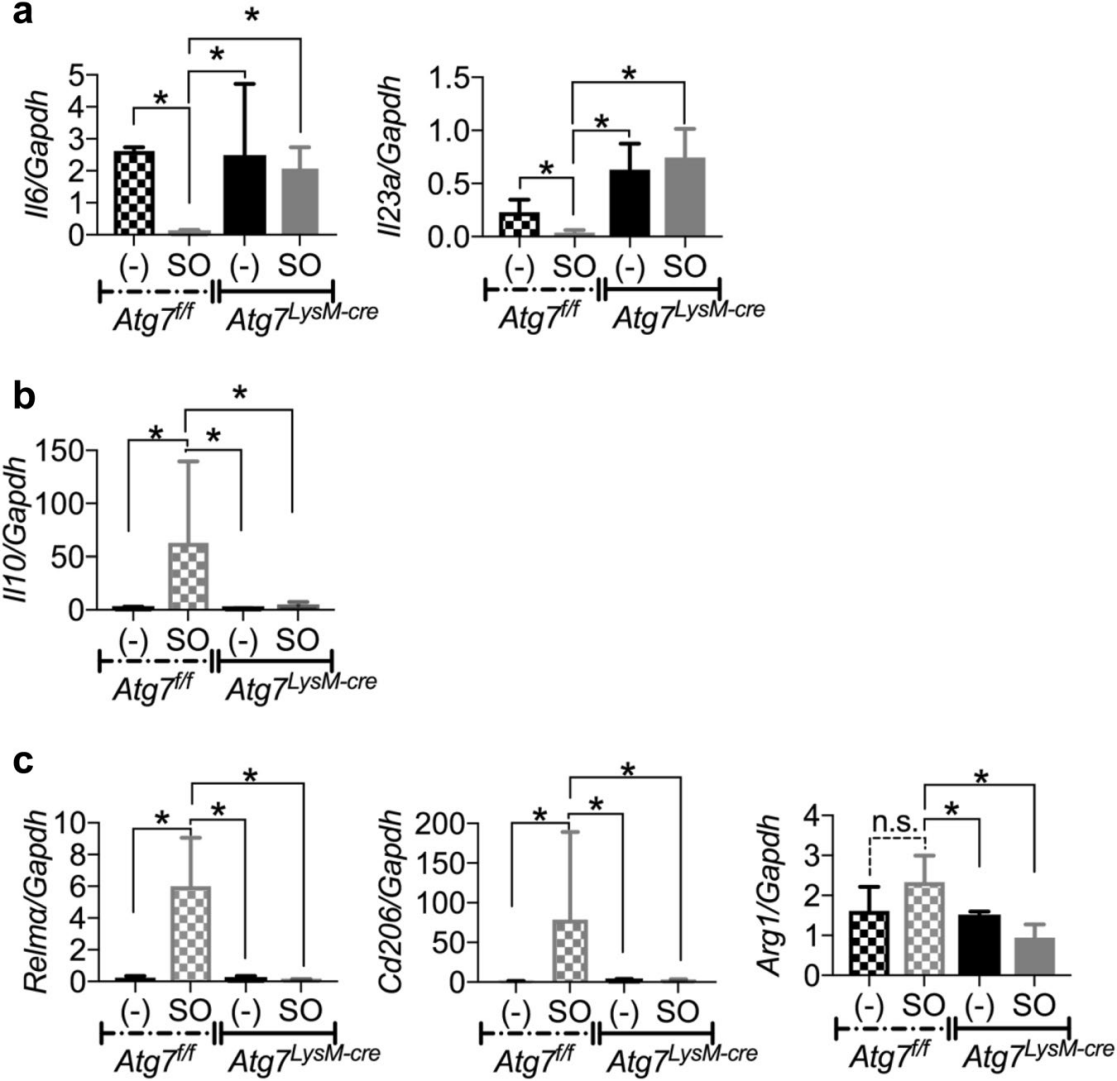
Promotion of Atg7-dependent autophagy by SO altered gene expression patterns in Mφ during DSS-induced colitis. (**a**–**c**) CD11b^+^ cells from the large intestinal lamina propria of *Atg7*^*f/f*^ and *Atg7*^*LysM-cre*^ mice administered 2%DSS with or without SO for 6 days were analyzed expression the indicated genes. Data are mean ± SD of three independent experiments. Data were analyzed by unpaired t-test, *p < 0.05. n.s., not significant.

To determine whether autophagy induced by SO in intestinal Mφ is responsible for prevention of intestinal inflammation, we analyzed innate myeloid cell-specific *Atg7-* deficient mice (*LysM-cre; Atg7*^*f/f*^ mice; hereafter called *Atg7* ^*LysM-cre*^ mice). During DSS administration, *Atg7*^*f/f*^ mice exhibited weight loss and shortened colon length, and severe intestinal pathology to similar levels to that in *Atg7* ^*LysM-cre*^ mice (Fig. 5a–c). In *Atg7*^*f/f*^ mice, co-administration of SO inhibited the weight loss, colon length shortening, epithelial disruption, and inflammatory cell infiltration. However, SO did not ameliorate DSS-induced colitis in *Atg7* ^*LysM-cre*^ mice. We further analyzed gene expression patterns in CD11b^+^ cells including Mφ isolated from the colons of *Atg7*^*f/f*^ and *Atg7* ^*LysM-cre*^ mice administered DSS with or without SO. In large intestinal CD11b^+^ cells of *Atg7* ^*f/f*^ mice, SO decreased the expression of pro-inflammatory cytokine including *Il6* and *Il23a*, whereas enhanced expression of an anti-inflammatory cytokine *Il10* (Fig. 6a,b). However, these alterations were not observed in *Atg7* ^*LysM-cre*^ mice. In addition, CD11b^+^ cells from the colon of *Atg7*^*f/f*^ mice highly expressed *Relma*, *Cd206*, and *Arg1*, which are maker genes of M2Mφ, a subset of anti-inflammatory Mφ, compared to those in *Atg7* ^*LysM-cre*^ mice (Fig. 6c). Moreover, we observed that SO treatment induced the expression of *Il10*, *Relma*, *Cd206*, and *Arg1* in BMMφ (Supplementary Fig. S5). We further analyzed contribution of SO to development of tissue-resident CX_3_CR1^high^ Mφ possessing anti-inflammatory properties. There were no differences in the numbers of CX3CR1^high^ CD11b^+^ Mφ and their precursor Ly6C^+^ CD11b^+^ monocytes between *Atg7*^*f/f*^ and *Atg7* ^*LysM-cre*^ mice (Supplementary Fig. S6a,b), suggesting that SO did not affect differentiation of CX3CR1^high^ CD11b^+^ Mφ during DSS-induced colitis. These findings demonstrate that SO ameliorates intestinal inflammation by providing anti-inflammatory profiles to Mφ via an autophagy-dependent mechanism.

### Tannins attenuate colitis by inducing Atg7-dependent Mφ autophagy

Among SO components, tannins including catechin acid (CA), ellagic acid (EA), and gallic acid (GA) and saponins including ziyuglycoside II (ZY) have been demonstrated to suppress inflammation in several tissues^10–12^. To determine whether these compounds prevent intestinal inflammation, mice were administered CA, EA, GA, and ZY equivalent to amounts found in SO during DSS-induced colitis. Single administration of each compound did not suppress large intestinal inflammation (Supplementary Fig. 7). However, a mixture of the four compounds (Mix_4_) led to reduced intestinal pathology to similar levels to that by SO.

**Figure 7:**
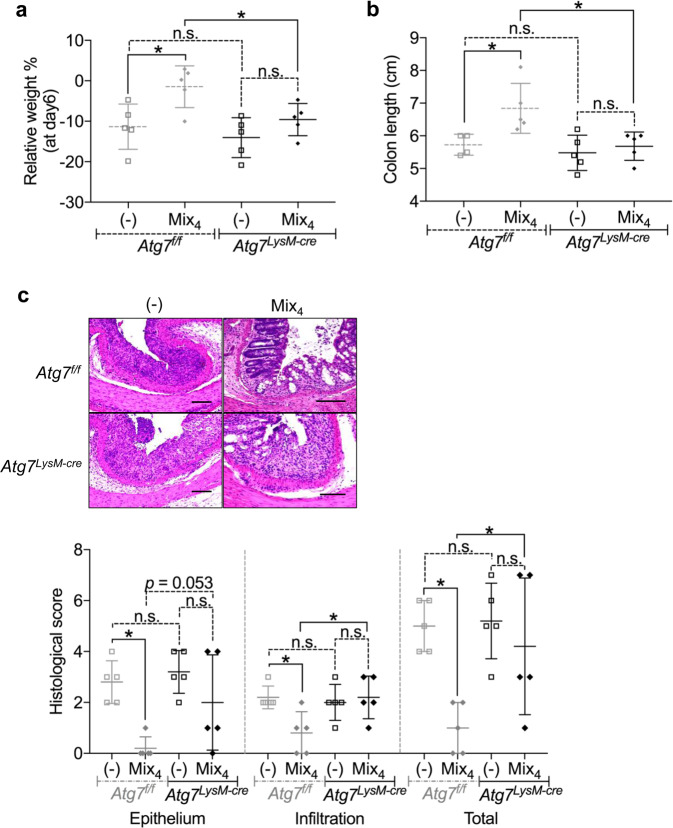
Minimal therapeutic effect of the mixture of SO components including CA, GA, EA, ZY (Mix_4_) on suppression of DSS-induced colitis in *Atg7*^*LysM-cre*^ mice. *Atg7*^*f/f*^ and *Atg7*^*LysM-cre*^ mice were administered 2% DSS with or without Mix_4_ for 7 days. (**a**) Weight changes, (**b**) colon length, (**c**) representative colon sections (upper), and histological score of the colon (bottom). Data are mean ± SD of five mice in each group. *p < 0.05. n.s., not significant. Bars; 100 μm. Data are of two independent experiments.

To determine whether induction of Atg7-dependent Mφ autophagy is involved in the prevention of intestinal inflammation by Mix_4_ shown in Supplementary Fig. S7, *Atg7*^*f/f*^ and *Atg7* ^*LysM-cre*^ mice were administered Mix_4_ during DSS-induced colitis. In *Atg7*^*f/f*^ mice, treatment with Mix_4_ diminished all clinical parameters including weight loss and colon shortening accompanied with less intestinal histopathology (Fig. 7a–c). In contrast, *Atg7* ^*LysM-cre*^ mice suffered from severe weight loss and colon shortening with profound infiltration of inflammatory cells and epithelial disruption even after Mix_4_ treatment. In this context, expression of marker genes of anti-inflammatory Mφ including *Relma*, *Cd206*, and *Arg1*, but not *Il10*, was upregulated in CD11b^+^ cells from the colon of *Atg7*^*f/f*^ mice following Mix_4_ administration, whereas expression of these genes was not altered in *Atg7* ^*LysM-cre*^ mice (Supplementary Fig. S8). BMMφ prepared from *Atg7* ^*f/f*^ and *Atg7* ^*LysM-cre*^ mice were cultured in the presence of SO, CA, EA, GA, and ZY for 24 hours and stained with CYTO-ID (Supplementary Fig. S9). Similar to SO, CA, EA, and GA, but not ZY, promoted autophagy in Mφ prepared from *Atg7* ^*f/f*^ mice, while it was not induced in *Atg7* deficient Mφ. These findings demonstrate that tannins contained in SO suppress intestinal inflammation coordinately through providing Mφ with anti-inflammatory profiles by inducing Atg7-dependent Mφ autophagy.

## Discussion

In this study, we highlighted that a herbal medicine SO induce Atg7-dependent autophagy, which providing Mφ with anti-inflammatory profiles, and thereby suppressing intestinal inflammation.

A previous study has defined that epithelial Atg7-dependent autophagy is dispensable for inhibition of DSS-induced colitis^45^. In accordance, we found that lack of Atg7 in intestinal epithelial cells did not affect the intestinal pathology during DSS-induced colitis without SO. In contrast, a study has demonstrated that epithelial cell-specific Atg7 deficiency leads to dysbiosis and thereby associating with development of more severe DSS-induced colitis^46^. This discrepancy seems to be explained by a differential composition of commensal bacteria between the studies, because more severe mucosal inflammation in the colon of epithelial cell-specific Atg7 mice was abrogated by antibiotics treatment^46^.

SO promoted epithelial autophagy in the large intestine during DSS-induced colitis. In *Atg7*^ΔIEC^ mice, mild colon shortening was observed even after SO treatment, although infiltration of inflammatory cells drastically reduced. A study has recently demonstrated that inappropriate activation of mesenchymal stromal cells contributes to the pathogenesis of DSS-induced colitis and human IBD^47^. Thus, there is a possibility that SO-mediated reinforcement of mucosal barrier integrity through activation of Atg7-dependent epithelial autophagy links to regulation of activation of non-hematopoietic lamina propria cells via an unknown mechanism, which may associate with less clinical parameters including colon shortening during DSS-induced colitis. In addition to the Atg5/Atg7-conventional pathway, autophagy occurs via the Atg5/Atg7-independent alternative pathway, which is regulated by Ulk1 and beclin 1^48^. We cannot exclude the possibility that SO suppresses intestinal inflammation by promoting epithelial autophagy through activation of the Atg5/Atg7-independent alternative pathway. Thus, it would be interesting to identify whether SO activates Ulk1/beclin 1-mediated autophagy in the inflammatory conditions and alleviate intestinal inflammation.

A previous report has demonstrated that there is no difference in reduction of body weight during DSS administration between *Atg7*^*f/f*^ and *Atg7*^*LysM-cre*^ mice^42^. In *Atg7*^*f/f*^ mice, body weight gain was seen within 3 days following the completion of DSS administration, whereas *Atg7*^*LysM-cre*^ mice kept their weight loss^42^. These findings suggest that Atg7-dependent autophagy in Mφ may be essential for promotion of tissue repair and regeneration in the intestine. As previously reported^42^, we found that there was no difference in the severity of intestinal inflammation between *Atg7*^*f/f*^ and *Atg7*^*LysM-cre*^ mice during DSS administration. In this context, SO-mediated enhancement of Atg7-dependent autophagy in Mφ led to suppression of intestinal pathology. In the current study, intestinal Mφ and BMMφ treated with SO acquired anti-inflammatory profiles that are similar to M2Mφ, such as upregulated the expression of *Arg1*, *Cd206*, and *Relma*. M2Mφ are known to have ability to inhibit inflammation and encourage tissue repair^49^. Previous studies have reported that lack of Atg5/Atg7-dependent autophagy in Mφ results in reduction of M2Mφ polarization in the liver and fat tissue^50,51^. In addition, a study has shown that CD5L, a secreted glycoprotein, induce M2Mφ through Atg7-dependent induction of Id3 expression^52^. Therefore, SO may prevent intestinal inflammation by accelerating M2Mφ polarization by facilitating Atg7-depedent autophagy. In the current study, co-administration of SO led to amelioration of DSS-induced intestinal inflammation, whereas pre-treatment with SO was ineffective. In the intestine, monocyte-derived CX_3_CR1^intermediate^ CD11b^+^ cells are increased during inflammation^35,36^. Thus, SO may provide these cells with M2Mφ-like phenotypes.

SO treatment led enhanced expression of *Il10* in colonic CD11b^+^ cells including Mφ of *Atg7*^*f/f*^ mice, but not *Atg7* ^*LysM-cre*^ mice. In the intestinal mucosa, expression of a subset of pro-inflammatory cytokines in Mφ is inhibited through IL-10-dependent activation of transcription factor Stat3, linking to prevention of intestinal inflammation^53–55^. In addition, a previous study has shown that intestinal Mφ-derived IL-10 is needed for maintenance of Foxp3^+^ Treg cells^56^. However, Mix_4_ did not promote *Il10* expression in colonic CD11b^+^ cells, although effectively ameliorating DSS-induced colitis. These findings suggest that upregulation of *Il10* expression in Mφ may be not required for SO-mediated prevention of intestinal inflammation.

In the current study, single administration of 15 μg/ml of CA, GA, EA, or ZY equivalent to amounts contained in 1 mg/ml of SO did not show a therapeutic effect during DSS administration, while a mixture of the four compounds suppressed intestinal inflammation through activation of Atg7-dependent Mφ autophagy. Among these compounds, CA, EA, and GA, which are categorized as tannin, induced Atg7-dependent autophagy in BMMφ, while ZY that is categorized as saponin did not. These findings raise the possibility that the combined total quantity of tannins within SO may be essential for suppression of intestinal inflammation. Thus, it would be important to analyze whether single-administration of higher-dose CA, GA, or EA is effective to prevent colitis in the future. Sanguiin H-1 to 11^57–59^, 1,4,6-Tri-*O*-galloyl-β-d-glucopyranose, 3,3′,4′-trimethylellagic acid^60^, and hamamelitannin^57^ that comprise the group of tannins are contained in SO. Thus, it would be interesting to examine whether these compotes are involved in SO-mediated inhibition of intestinal inflammation.

In IBD patients, intestinal CD14^+^ Mφ show facilitated production of inflammatory mediators including IL-6 and IL-23 in response to commensal bacteria^44,61^. Thus, it would be important to examine whether SO downregulates production of these cytokines in human intestinal Mφ by providing anti-inflammatory profiles to give insights for the development of novel therapeutic intervention with either SO or its components for IBD.

## Methods

### Mice

C57BL/6J mice were obtained from Clea Japan, Inc. (Tokyo, Japan). Mice carrying a loxP-flanked Atg7 allele (*Atg7*^*flox*^) were kindly provided by Prof. M. Komatsu (Juntendo University) and Dr. K. Tanaka (Tokyo Metropolitan Institute of Medical Science). Seven-week-old female mice were used for the experiments. Mouse experiments were approved by the Animal Research Committee of the Institute of Experimental Animal Sciences Faculty of Medicine (Osaka University, Osaka, approval no. 27-099-000). All methods were carried out in accordance with guidelines and regulations of Animal Research Committee of the Institute of Experimental Animal Sciences Faculty of Medicine in Osaka University. All mice were fed a standard diet and maintained in routine light-dark cycles, and housed in specific pathogen-free conditions.

### Reagents

Immunohistochemistry was performed following antibodies anti-rabbit LC3B (dilution 1:200, Abcam Plc., Cambridge, UK), anti-rabbit Alexa Flour 647 (Thermo Fisher Scientific K.K., Massachusetts, USA). Apoptosis of intestinal epithelial cell was detected using the ApopTag Fluorescein *In situ* Apoptosis Detection Kit (Merck KGaA, Darmstadt, Germany). Autophagic structures and nucleus were determined using the CYTO-ID detection kit contains Hoechst33342 (Enzo Biochem Inc., New York, USA). Images of staining samples were taken by using BZ-X 700 (Keyence Corporation, Osaka, Japan) or FV1200 (BX) (Olympus Corporation, Tokyo, Japan).

### Flow cytometry

The following antibodies were purchased from Becton, Dickinson and Company (BD) (Franklin Lakes, New Jersey, USA): 7-AAD, anti-mouse CD16/32 (clone 2.4.G2), PE-Cy7-conjugated anti-mouse-Ly-6C (clone AL-21), PE- or FITC-conjugated anti-mouse CD11b (clone M1/70), PE-conjugated anti-mouse-SiglecF (clone E50-2440) and anti-mouse Ly6G (clone 1A8). Pacific blue-conjugated anti-mouse CD45 (clone 30-F11) APC-conjugated anti mouse F4/80 (clone BM8) and PE-conjugated anti-CX3CR1 (clone SA011F11) antibodies were purchased from BioLegend, Inc. (San Diego, California, USA). PE-Cy7-conjugated anti-mouse CD11b (clone M1/70) and PE-conjugated anti mouse MHC ClassII (clone M5/114.15.2) was purchased from Bay Bioscience Co., Ltd. (Hyogo, Japan). Flow cytometric analysis was performed with a FACSCanto II flow cytometer (BD) with FlowJo software (FlowJo Llc., Ashland, Oregon, USA). The instrumental compensation was set in each experiment using four-color stained samples.

### Identification of autophagy inducers with high-throughput assay system

1 × 10^3^ GFP-LC3-expressing mouse embryonic fibroblasts were cultured in each well of 96-well plate for 16 hours and subsequently treated with each substance included in a natural compound library. After 5 hours, the area of GFP-LC3 puncta was measured with Incell Analyzer 1000 (GE Healthcare UK Ltd., England).

### DSS-induced colitis

Mice were administered 2% DSS (160110, molecular weight 36,000–50,000; MP Biomedicals Inc., Ohio, USA; lot number Q3526) in their drinking water for 7 days. Their weight was adjusted to 16–19 g on the day of the experiment (day 0). Weight and water consumption were measured every day. Colon length was measured 7 days after starting DSS administration. The colons were fixed in 10% formalin over 24 hours and embedded in paraffin. 3.5-µm-thick sections were stained with hematoxylin and eosin (H&E). Images of hematoxylin and eosin staining were taken using BZ-X 700 (Keyence). The histological scores were analyzed according to the method previously reported^62^.

Intestinal permeability was assessed using FITC-dextran (molecular weight; 4000, Chondrex Inc., DC, USA) according to the manufacturer’s instructions. The concentration of FITC-dextran in plasma was quantified with a fluorescent plate reader (SH-9000, Corona Electric Co., Ltd, Ibaragi, Japan).

### Isolation of large intestinal lamina propria cells

Large intestinal lamina propria cells were isolated using previously described protocol^63^. Large intestinal lamina propria CD11b^+^ cells were collected using MACS CD11b magnetic beads (Miltenyi Biotec K.K., Bergisch Gladbach, Germany) according to the manufacturer’s protocol.

### Preparation of the cells

To prepare BMMφ, bone marrow cells were isolated from mouse femurs and tibias, passed through a nylon mesh, and cultured in RPMI1640 (Nacarai Tesque Inc., Kyoto Japan) medium containing 10% fetal bovine serum (FBS; GE Healthcare Life Sciences, Ilinois, USA), penicillin (10,000 unit/ml), and streptomycin (10,000 µg/ml), and 30% L929 cell culture supernatant for 7 days.

### Preparation of SO and components including SO

SO roots was purchased from Tochimoto Tenkaido Co., Ltd. (Osaka, Japan). The ingredients were extracted by boiling in 10 times water for 1 hour. After filtration, the extracted components were lyophilized to powder. 0.01, 0.1, and 1 mg/mL of SO extract was co-administered with DSS orally to mice. CA, GA, EA and ZY were purchased from Namiki Shoji Co., Ltd. (Tokyo, Japan). The concentrations of these components in SO were calculated, as reported previously^9^ and 15 µg/ml of CA, GA, EA, and ZY in drinking water were co-administered with DSS to mice.

### Western blotting

Cell lysate and tissue sample were dissolved in RIPA buffer (Thermo) with protease inhibitor (Thermo) and phosphatase inhibitor (Thermo). Immunoblotting was performed using anti-mouse LC3B antibody (dilution 1:1000), anti- mouse beta-actin (dilution 1:1000, Sigma-Aldrich Co. Llc, SLE, U.S.A) and anti- mouse GAPDH (dilution 1:2000, Abcam).

### Quantitative real-time polymerase chain reactions (qRT-PCR) analysis

Total RNA was extracted from large intestinal lamina propria cells and epithelial cells using RNeasy Mini Kit (Qiagen K.K., California, USA). The RNA was reverse-transcribed into first-strand complementary DNA using the High-Capacity RNA-to-cDNA Kit (Thermo) according to the manufacture’s protocol. qRT**-**PCR was performed on an ABI Prism 7900HT sequence detection system (Applied Biosystems (Thermo)) using Thunderbird Probe qPCR Mix (Toyobo Co., Ltd, Osaka, Japan). The amplification conditions were 95 °C (10 min), 40 cycles of 95 °C (15 s) and 60 °C (60 s). All values were normalized to the expression of *Gapdh*, encoding glyceraldehyde-3-phosphate dehydrogenase, and the fold difference in expression relative to that of *Gapdh* is shown. All real-time qRT-PCR reactions were performed in triplicate.

### Statistical analysis

All experiments were performed using randomly assigned mice. All statistical analyses were performed using JMP Pro 13. (SAS Institute Inc., North Carolina, USA). Dunnett’s test for multiple comparisons, and unpaired, two-tailed Student’s t tests for comparisons between two groups were used to assess statistical significance. P values < 0.05 was considered to indicate a significant difference.

## Supplementary information

Supplementary Information.

Supplementary Information 2.
