# Supplementary material for: Sanguisorba officinalis L. derived from herbal medicine prevents intestinal inflammation by inducing autophagy in macrophages
Source: Sci Rep. 2020 Jun 19;10:9972. doi: 10.1038/s41598-020-65306-4 (PMC7305163; doi:10.1038/s41598-020-65306-4)
Supplement: Supplementary file 1 — Supplementary Information. [file 41598_2020_65306_MOESM1_ESM.pptx]

## Slide 1
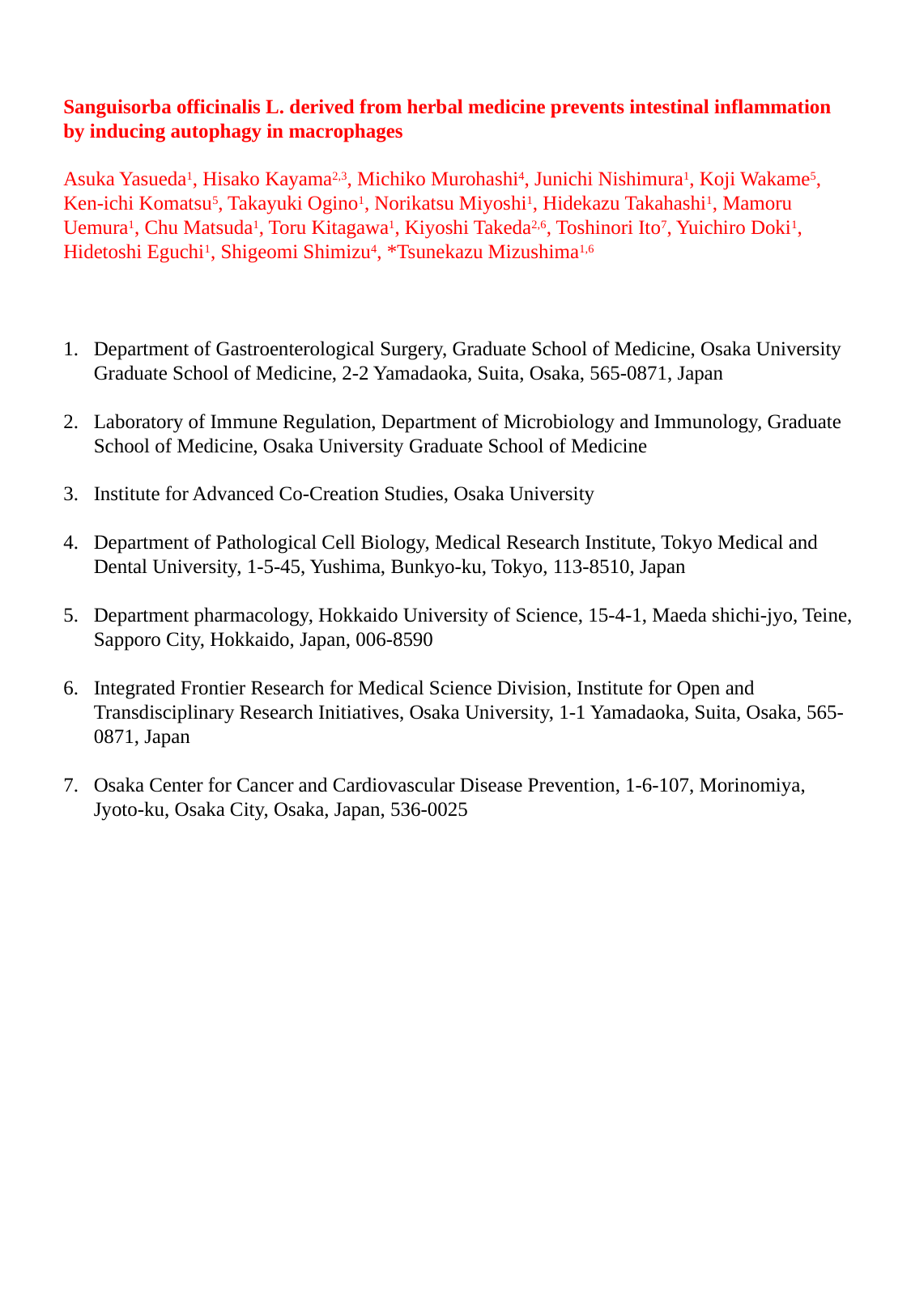

Sanguisorba officinalis L. derived from herbal medicine prevents intestinal inflammation by inducing autophagy in macrophages
Asuka Yasueda1, Hisako Kayama2,3, Michiko Murohashi4, Junichi Nishimura1, Koji Wakame5, Ken-ichi Komatsu5, Takayuki Ogino1, Norikatsu Miyoshi1, Hidekazu Takahashi1, Mamoru Uemura1, Chu Matsuda1, Toru Kitagawa1, Kiyoshi Takeda2,6, Toshinori Ito7, Yuichiro Doki1, Hidetoshi Eguchi1, Shigeomi Shimizu4, *Tsunekazu Mizushima1,6
Department of Gastroenterological Surgery, Graduate School of Medicine, Osaka University Graduate School of Medicine, 2-2 Yamadaoka, Suita, Osaka, 565-0871, Japan
Laboratory of Immune Regulation, Department of Microbiology and Immunology, Graduate School of Medicine, Osaka University Graduate School of Medicine
Institute for Advanced Co-Creation Studies, Osaka University
Department of Pathological Cell Biology, Medical Research Institute, Tokyo Medical and Dental University, 1-5-45, Yushima, Bunkyo-ku, Tokyo, 113-8510, Japan
Department pharmacology, Hokkaido University of Science, 15-4-1, Maeda shichi-jyo, Teine, Sapporo City, Hokkaido, Japan, 006-8590
Integrated Frontier Research for Medical Science Division, Institute for Open and Transdisciplinary Research Initiatives, Osaka University, 1-1 Yamadaoka, Suita, Osaka, 565-0871, Japan
Osaka Center for Cancer and Cardiovascular Disease Prevention, 1-6-107, Morinomiya, Jyoto-ku, Osaka City, Osaka, Japan, 536-0025

## Slide 2
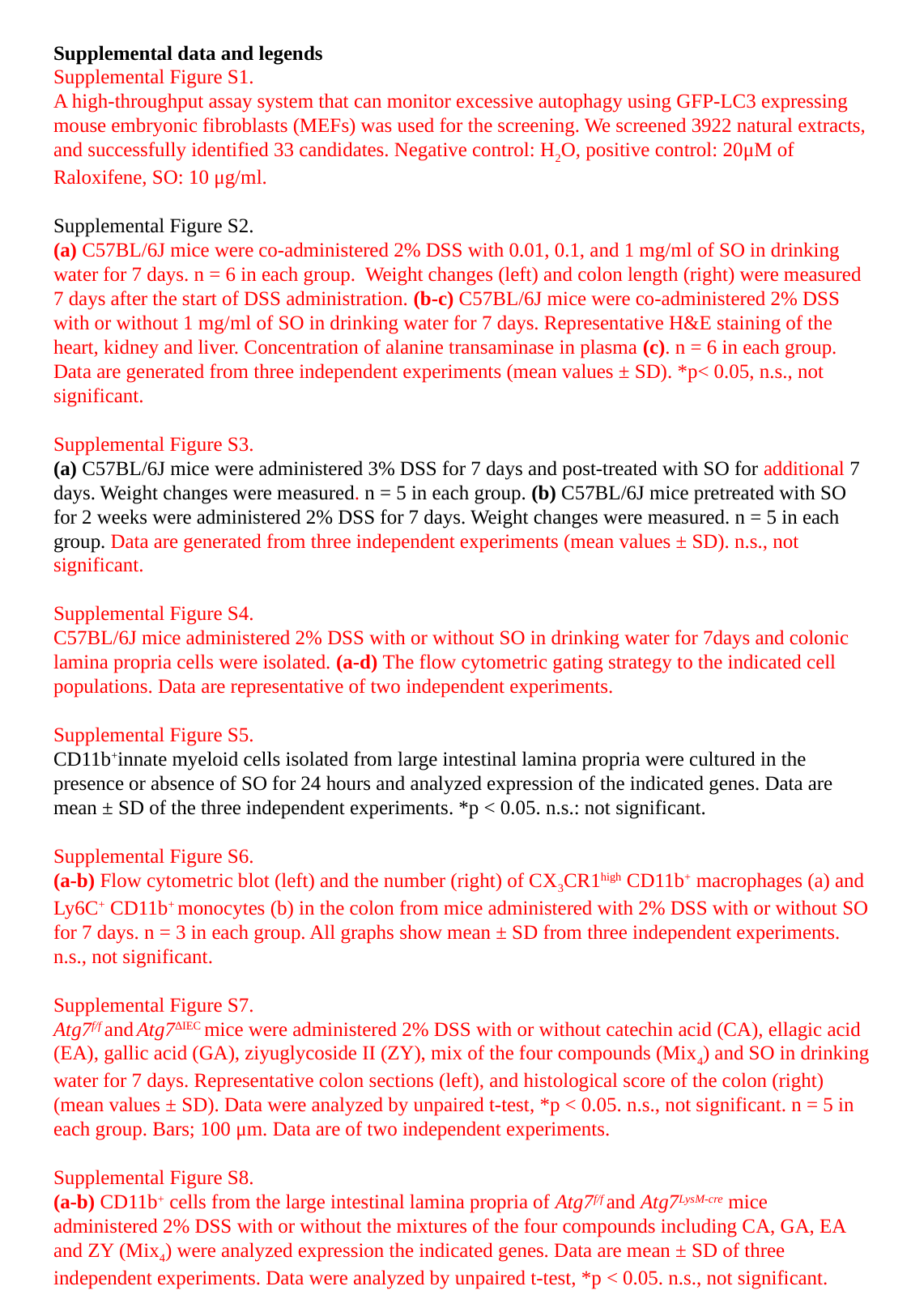

Supplemental data and legends
Supplemental Figure S1.
A high-throughput assay system that can monitor excessive autophagy using GFP-LC3 expressing mouse embryonic fibroblasts (MEFs) was used for the screening. We screened 3922 natural extracts, and successfully identified 33 candidates. Negative control: H2O, positive control: 20μM of Raloxifene, SO: 10 μg/ml.
Supplemental Figure S2.
(a) C57BL/6J mice were co-administered 2% DSS with 0.01, 0.1, and 1 mg/ml of SO in drinking water for 7 days. n = 6 in each group. Weight changes (left) and colon length (right) were measured 7 days after the start of DSS administration. (b-c) C57BL/6J mice were co-administered 2% DSS with or without 1 mg/ml of SO in drinking water for 7 days. Representative H&E staining of the heart, kidney and liver. Concentration of alanine transaminase in plasma (c). n = 6 in each group. Data are generated from three independent experiments (mean values ± SD). *p< 0.05, n.s., not significant.
Supplemental Figure S3.
(a) C57BL/6J mice were administered 3% DSS for 7 days and post-treated with SO for additional 7 days. Weight changes were measured. n = 5 in each group. (b) C57BL/6J mice pretreated with SO for 2 weeks were administered 2% DSS for 7 days. Weight changes were measured. n = 5 in each group. Data are generated from three independent experiments (mean values ± SD). n.s., not significant.
Supplemental Figure S4.
C57BL/6J mice administered 2% DSS with or without SO in drinking water for 7days and colonic lamina propria cells were isolated. (a-d) The flow cytometric gating strategy to the indicated cell populations. Data are representative of two independent experiments.
Supplemental Figure S5.
CD11b+innate myeloid cells isolated from large intestinal lamina propria were cultured in the presence or absence of SO for 24 hours and analyzed expression of the indicated genes. Data are mean ± SD of the three independent experiments. *p < 0.05. n.s.: not significant.
Supplemental Figure S6.
(a-b) Flow cytometric blot (left) and the number (right) of CX3CR1high CD11b+ macrophages (a) and Ly6C+ CD11b+ monocytes (b) in the colon from mice administered with 2% DSS with or without SO for 7 days. n = 3 in each group. All graphs show mean ± SD from three independent experiments. n.s., not significant.
Supplemental Figure S7.
Atg7f/f and Atg7ΔIEC mice were administered 2% DSS with or without catechin acid (CA), ellagic acid (EA), gallic acid (GA), ziyuglycoside II (ZY), mix of the four compounds (Mix4) and SO in drinking water for 7 days. Representative colon sections (left), and histological score of the colon (right) (mean values ± SD). Data were analyzed by unpaired t-test, *p < 0.05. n.s., not significant. n = 5 in each group. Bars; 100 μm. Data are of two independent experiments.
Supplemental Figure S8.
(a-b) CD11b+ cells from the large intestinal lamina propria of Atg7f/f and Atg7LysM-cre mice administered 2% DSS with or without the mixtures of the four compounds including CA, GA, EA and ZY (Mix4) were analyzed expression the indicated genes. Data are mean ± SD of three independent experiments. Data were analyzed by unpaired t-test, *p < 0.05. n.s., not significant.

## Slide 3
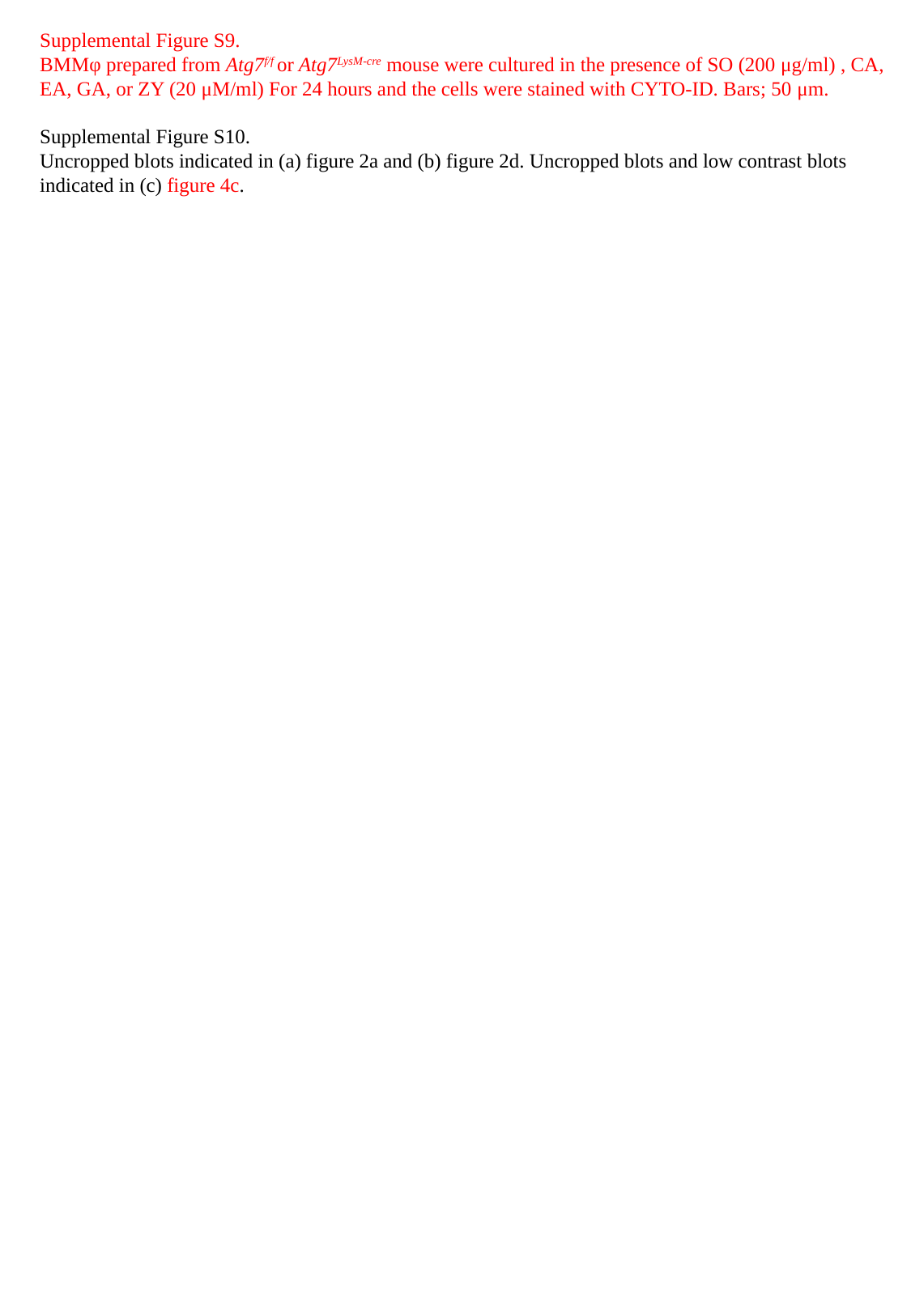

Supplemental Figure S9.
BMMφ prepared from Atg7f/f or Atg7LysM-cre mouse were cultured in the presence of SO (200 μg/ml) , CA, EA, GA, or ZY (20 μM/ml) For 24 hours and the cells were stained with CYTO-ID. Bars; 50 μm.
Supplemental Figure S10.
Uncropped blots indicated in (a) figure 2a and (b) figure 2d. Uncropped blots and low contrast blots indicated in (c) figure 4c.

## Slide 4
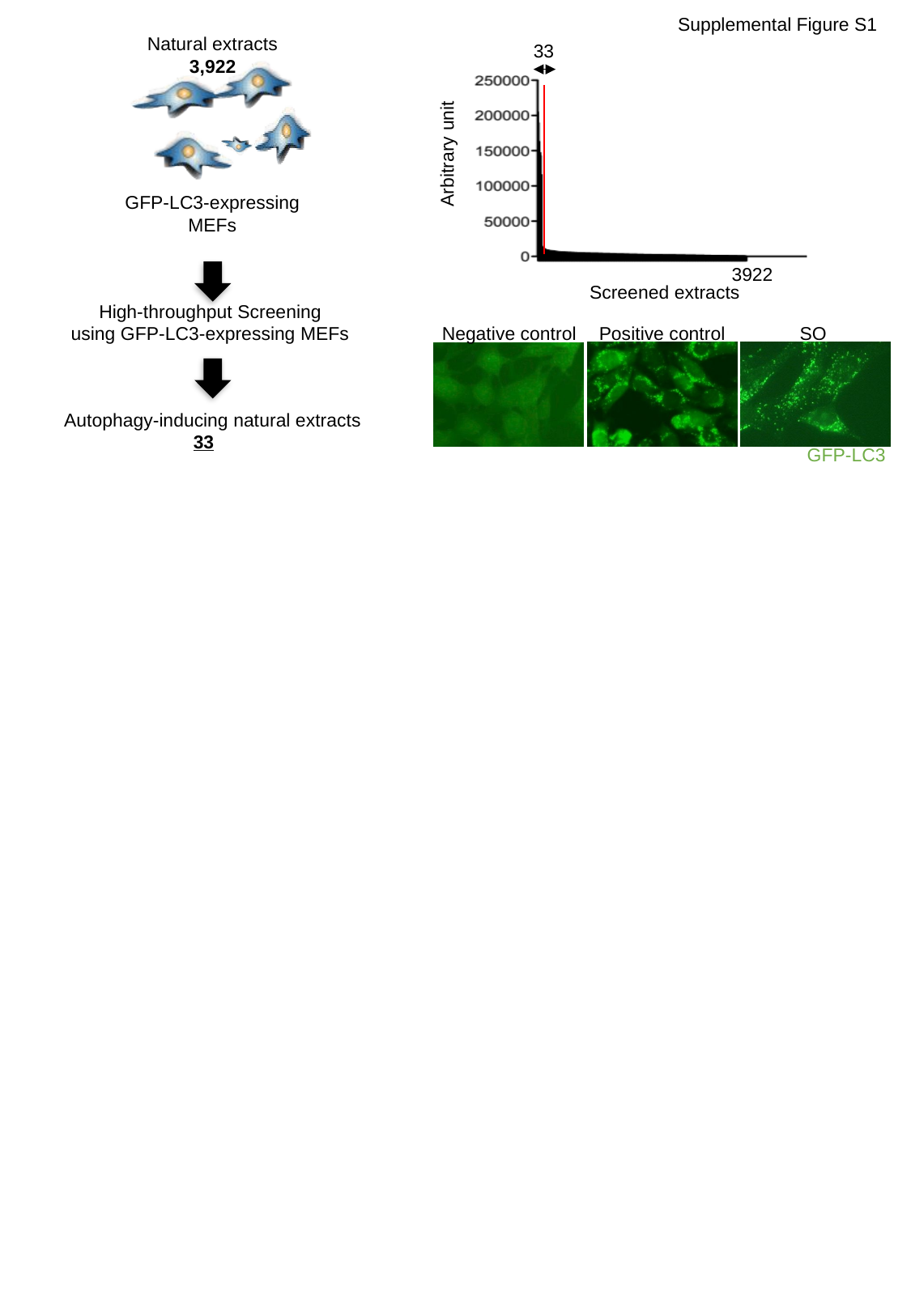

Supplemental Figure S1
Natural extracts
3,922
33
Arbitrary unit
3922
Screened extracts
GFP-LC3-expressing
MEFs
High-throughput Screening
using GFP-LC3-expressing MEFs
Positive control
SO
Negative control
Autophagy-inducing natural extracts
33
GFP-LC3

## Slide 5
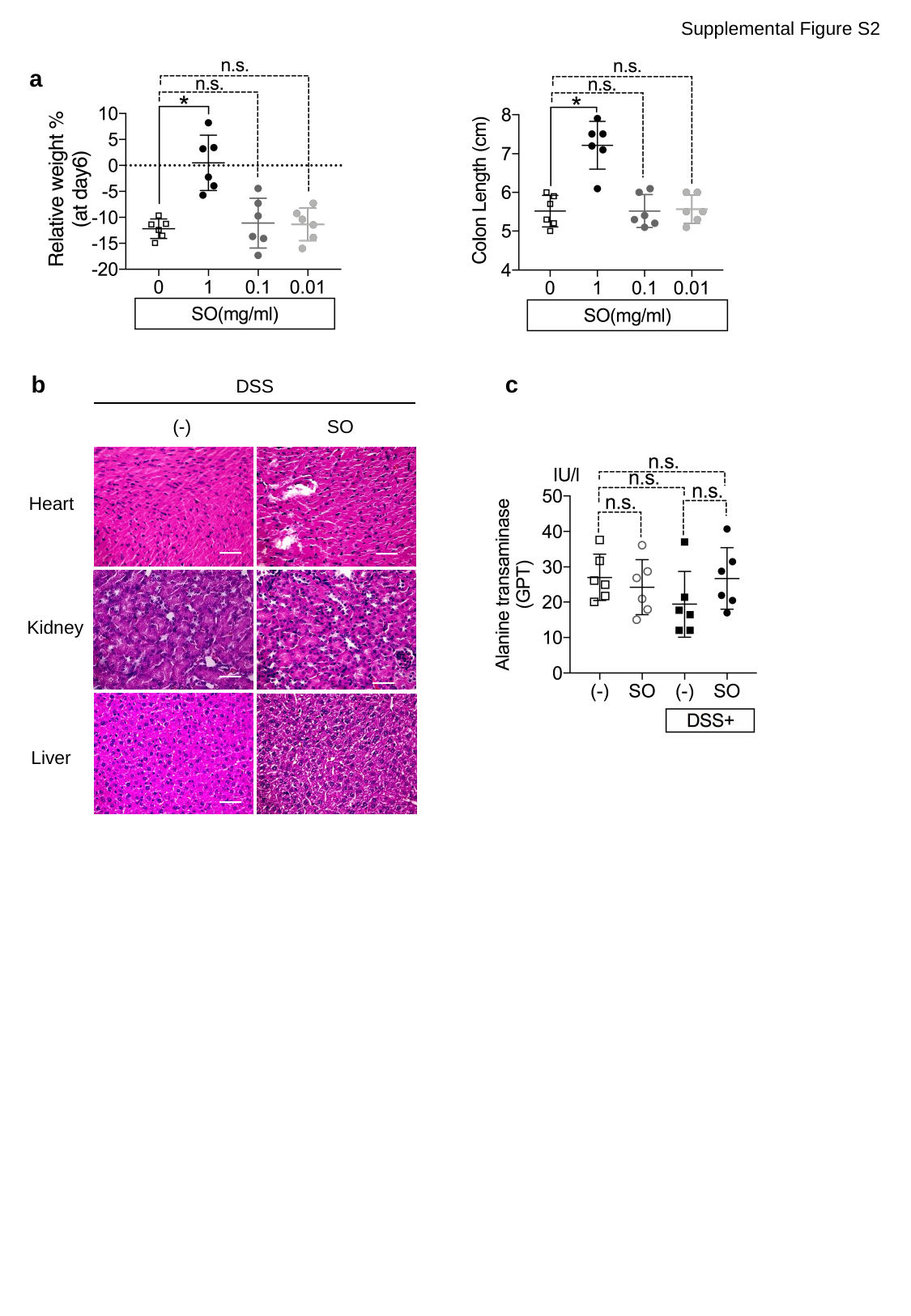

Supplemental Figure S2
a
b
c
DSS
SO
(-)
Heart
Kidney
Liver

## Slide 6
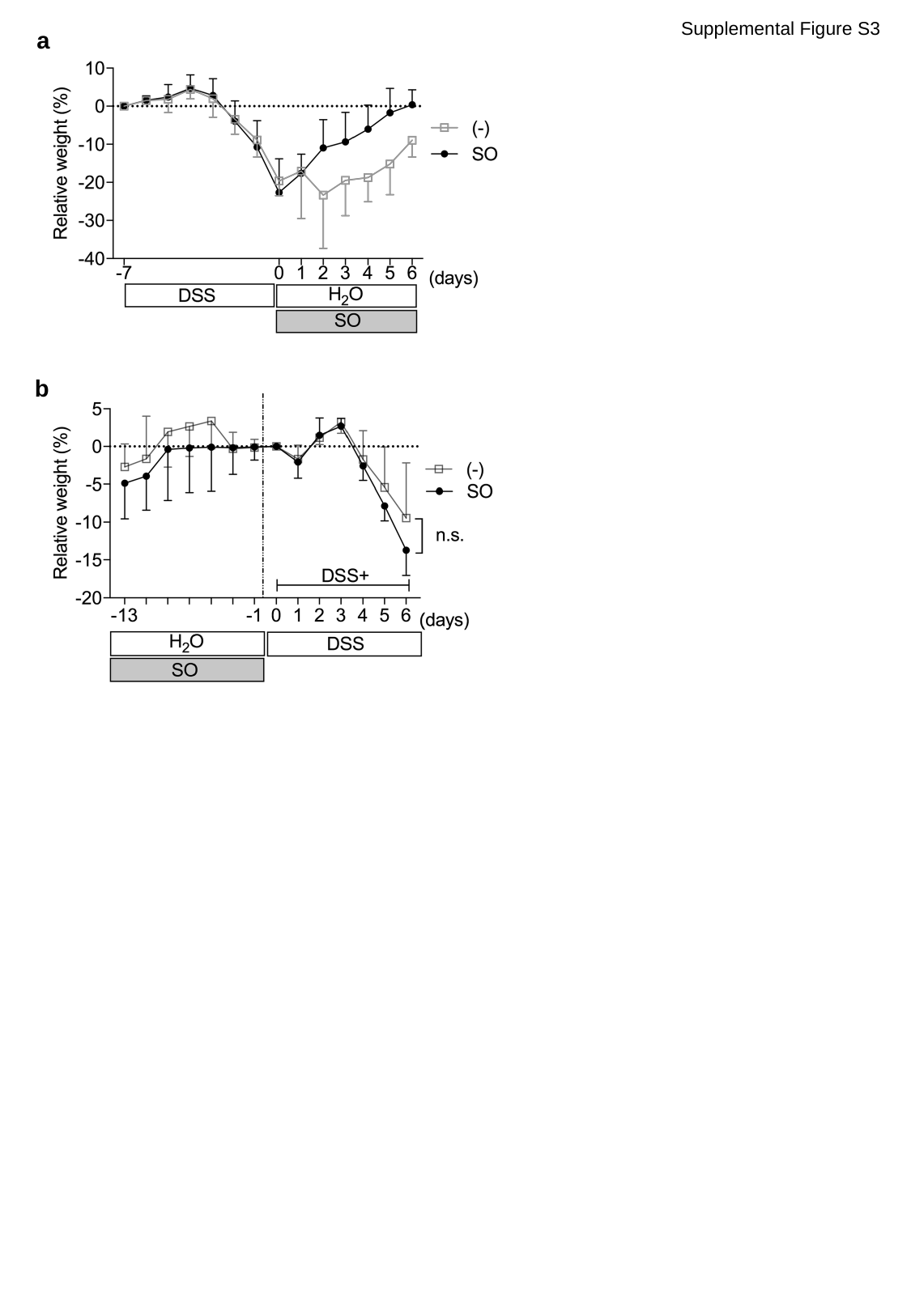

Supplemental Figure S3
a
b

## Slide 7
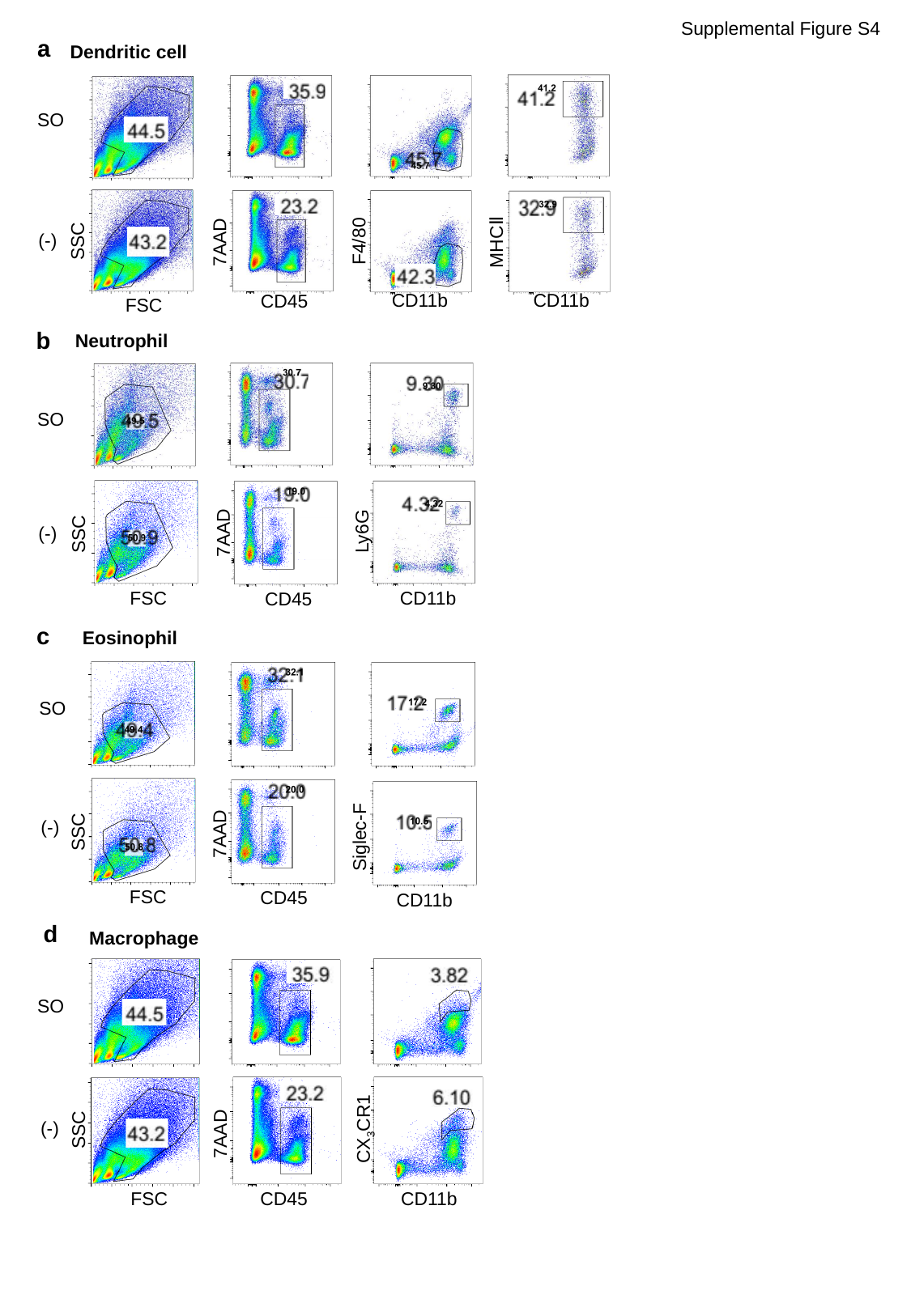

Supplemental Figure S4
a
Dendritic cell
SO
(-)
SSC
F4/80
7AAD
MHCⅡ
CD11b
CD11b
CD45
FSC
b
Neutrophil
SO
Ly6G
7AAD
(-)
SSC
FSC
CD11b
CD45
c
Eosinophil
SO
(-)
SSC
7AAD
Siglec-F
FSC
CD45
CD11b
d
Macrophage
SO
(-)
CX3CR1
SSC
7AAD
FSC
FSC
CD11b
CD45

## Slide 8
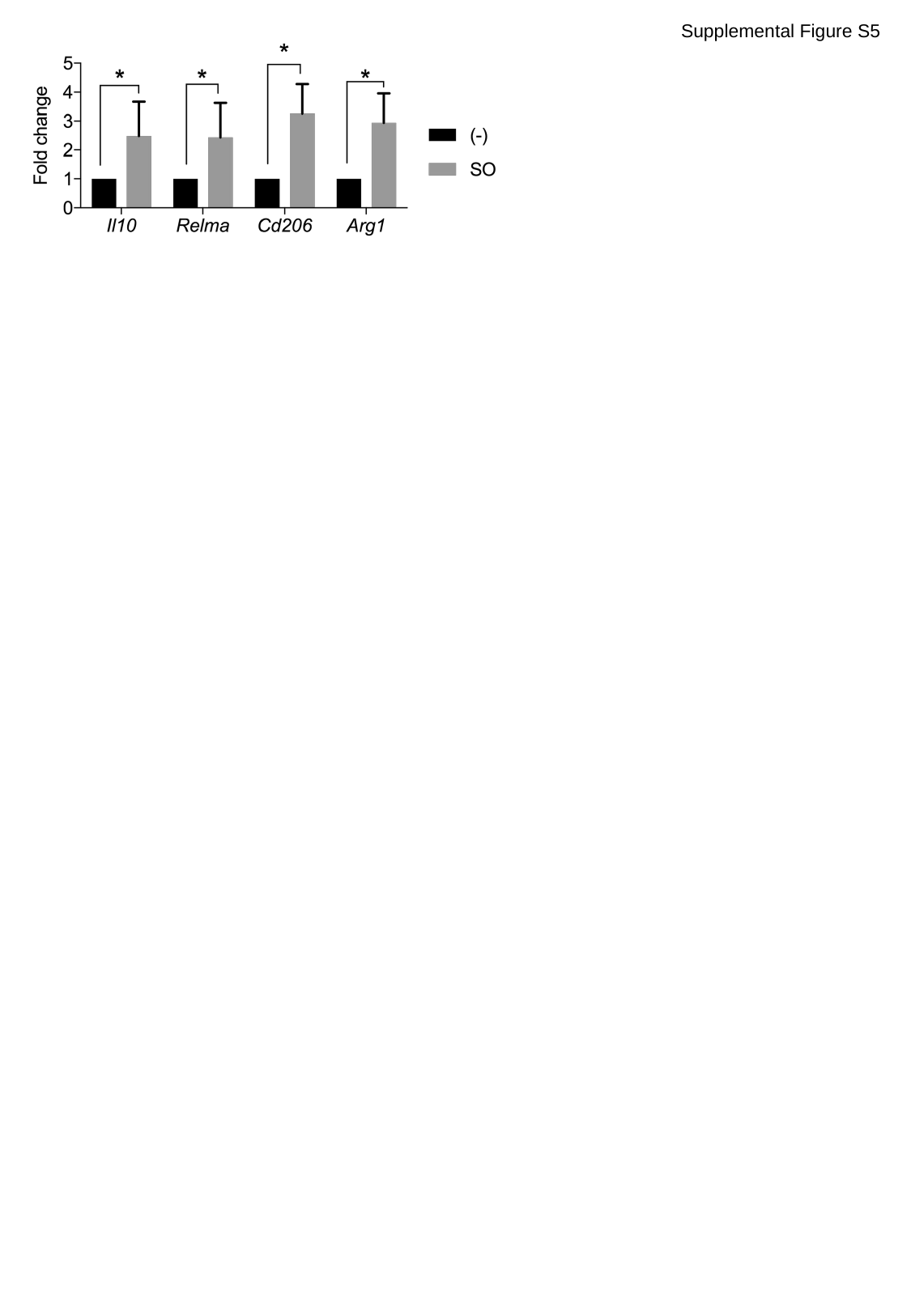

Supplemental Figure S5

## Slide 9
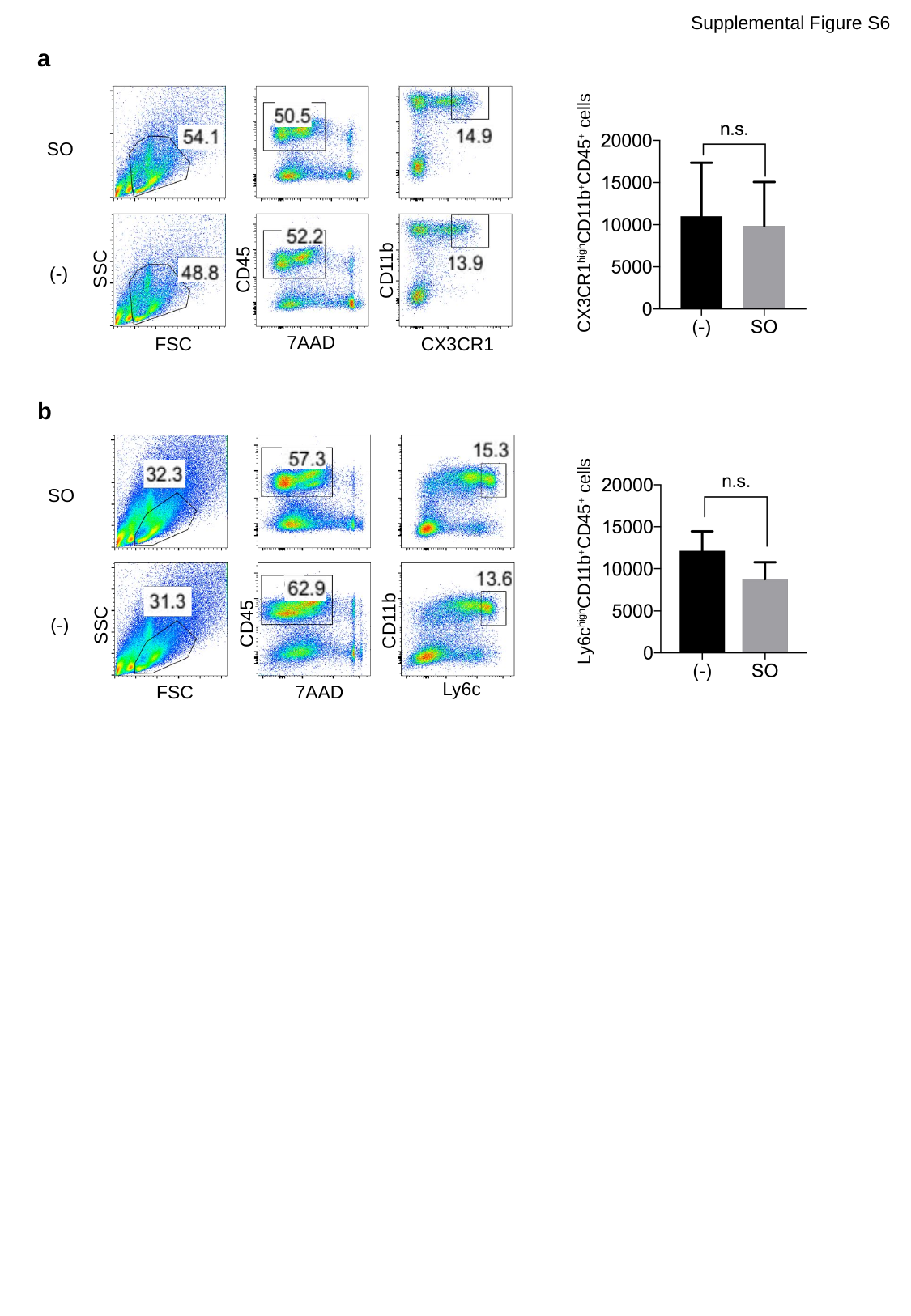

Supplemental Figure S6
a
SO
CX3CR1highCD11b+CD45+ cells
SSC
(-)
7AAD
FSC
CX3CR1
CD45
CD11b
b
SO
Ly6chighCD11b+CD45+ cells
CD11b
CD45
(-)
SSC
Ly6c
FSC
7AAD

## Slide 10
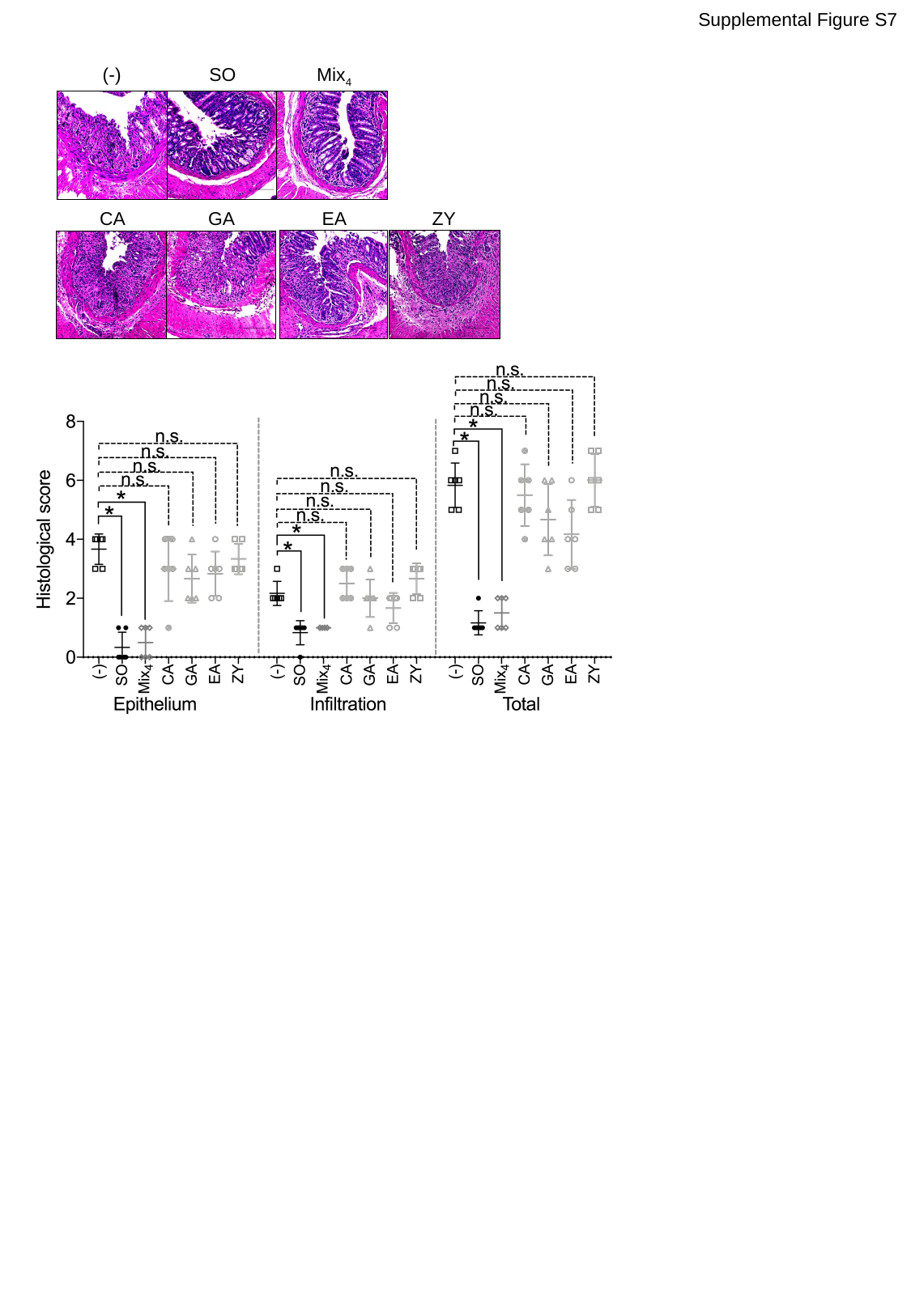

Supplemental Figure S7
(-)
SO
Mix4
CA
GA
EA
ZY

## Slide 11
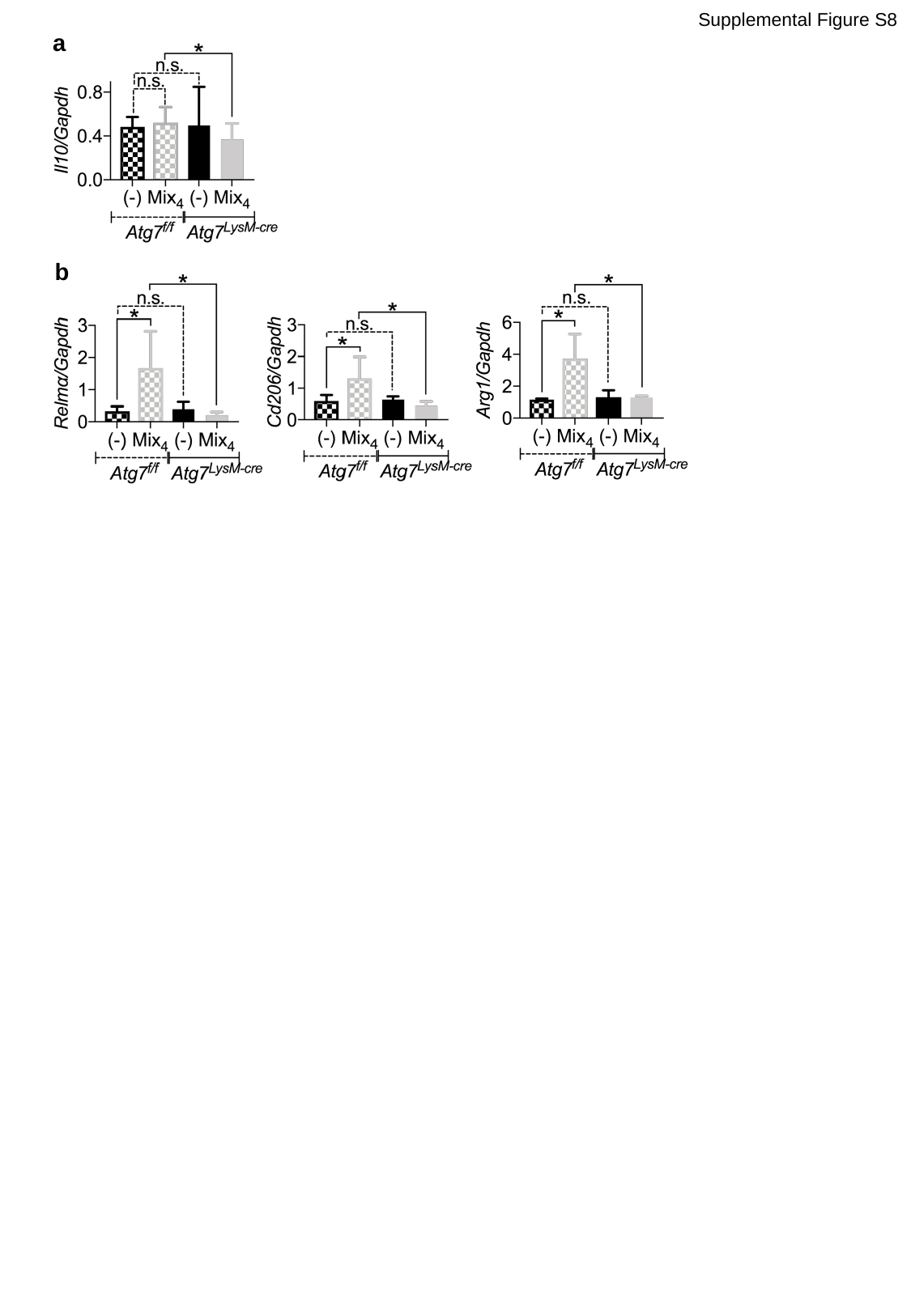

Supplemental Figure S8
a
b

## Slide 12
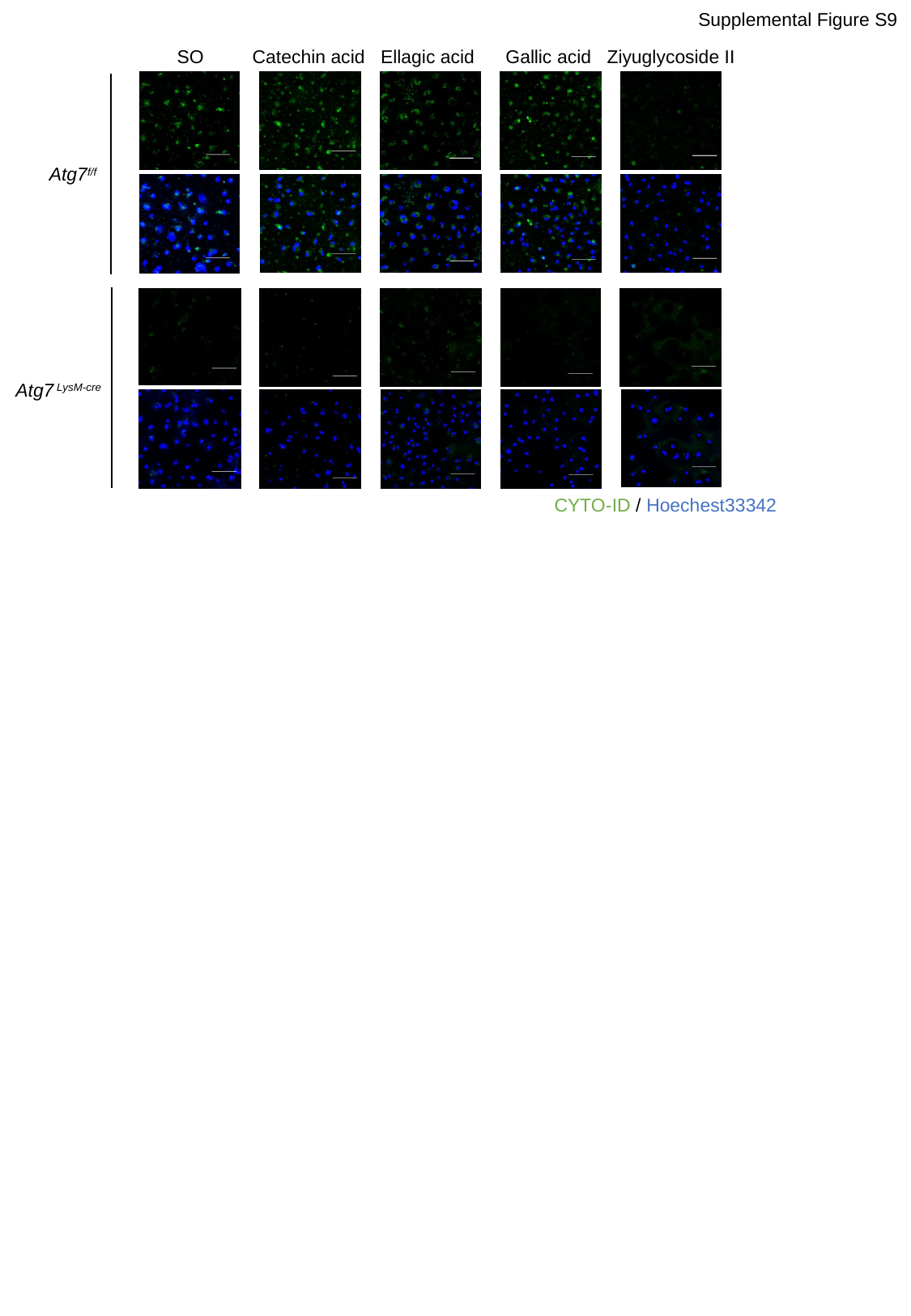

Supplemental Figure S9
SO
Catechin acid
Ellagic acid
Gallic acid
Ziyuglycoside II
CYTO-ID / Hoechest33342
Atg7f/f
Atg7 LysM-cre

## Slide 13
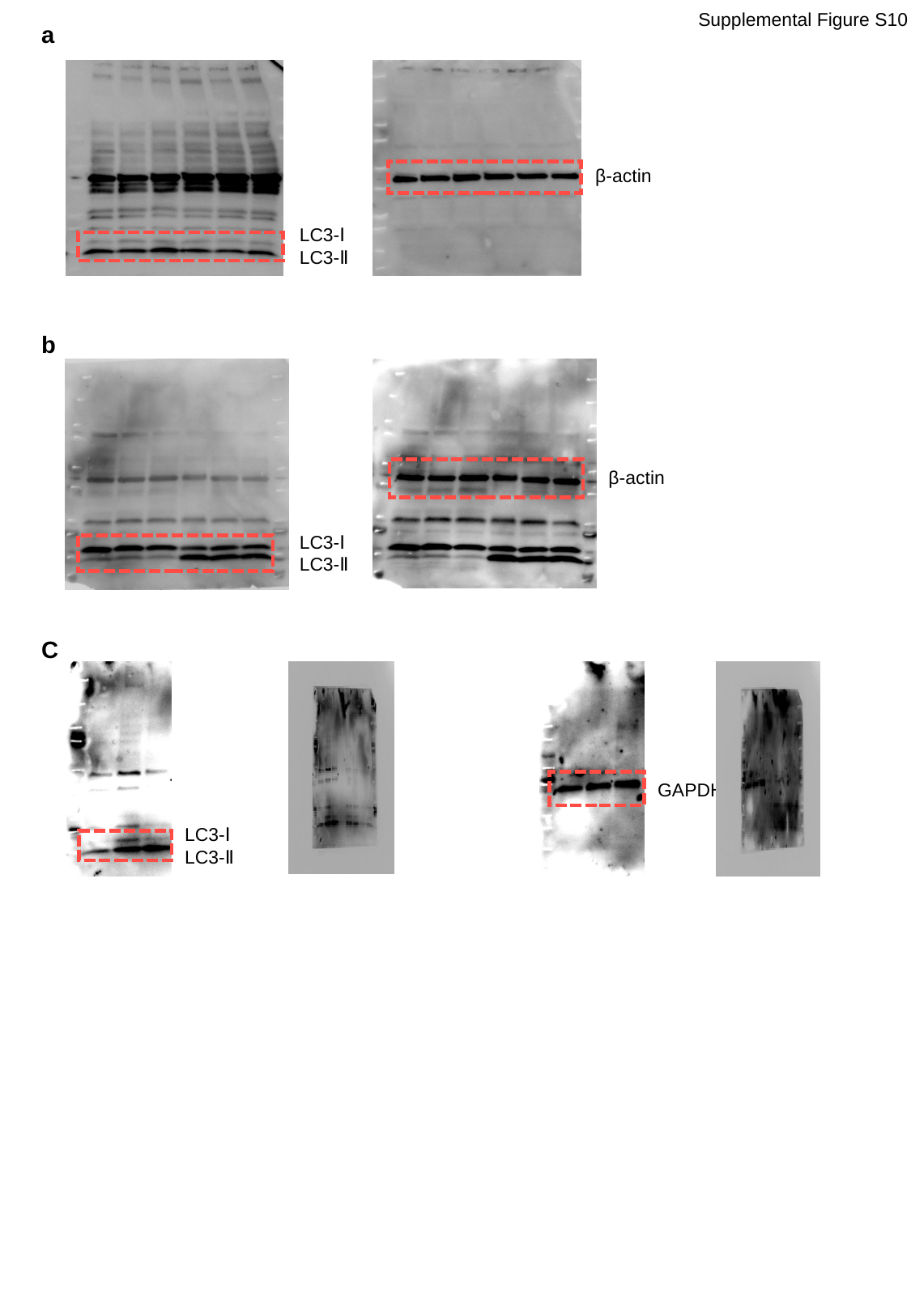

Supplemental Figure S10
a
β-actin
LC3-Ⅰ
LC3-Ⅱ
b
β-actin
LC3-Ⅰ
LC3-Ⅱ
C
GAPDH
LC3-Ⅰ
LC3-Ⅱ
