# Supplementary material for: Sanguisorba officinalis L. derived from herbal medicine prevents intestinal inflammation by inducing autophagy in macrophages
Source: Sci Rep. 2020 Jun 19;10:9972. doi: 10.1038/s41598-020-65306-4 (PMC7305163; doi:10.1038/s41598-020-65306-4)
Supplement: Supplementary file 2 — Supplementary Information 2. [file 41598_2020_65306_MOESM2_ESM.pdf]

Sanguisorba Officinalis L. derived from herbal medicine prevents intestinal inflammation by inducing autophagy in innate myeloid cells

Asuka Yasueda1, *Tsunekazu Mizushima1,2, Michiko Murohashi3, Hisako Kayama4,5, Junichi Nishimura1, Koji Wakame6, Ken-ichi Komatsu6, Takayuki Ogino1, Norikatsu Miyoshi1, Hidekazu Takahashi1, Mamoru Uemura1, Chu Matsuda1, Toru Kitagawa1, Kiyoshi Takeda2,4, Toshinori Ito7, Shigeomi Shimizu2, Yuichiro Doki1 and Hidetoshi Eguchi1

1. Department of Gastroenterological Surgery, Graduate School of Medicine, Osaka University Graduate School of Medicine, 2-2 Yamadaoka, Suita, Osaka, 565-0871, Japan
2. Integrated Frontier Research for Medical Science Division, Institute for Open and Transdisciplinary Research Initiatives, Osaka University, 1-1 Yamadaoka, Suita, Osaka, 565-0871, Japan
3. Department of Pathological Cell Biology, Medical Research Institute, Tokyo Medical and Dental University, 1-5-45, Yushima, Bunkyo-ku, Tokyo, 113-8510, Japan
4. Laboratory of Immune Regulation, Department of Microbiology and Immunology, Graduate School of Medicine, Osaka University Graduate School of Medicine
5. Institute for Advanced Co-Creation Studies, Osaka University
6. Department pharmacology, Hokkaido University of Science, 15-4-1, Maeda shichi-jyo, Teine, Sapporo City, Hokkaido, Japan, 006-8590
7. Osaka Center for Cancer and Cardiovascular Disease Prevention, 1-6-107, Morinomiya, Jyoto-ku, Osaka City, Osaka, Japan, 536-0025

Supplemental table

Supplementary Table S1. Sequences of the primers used for qRT-PCR

Gene	Forward primer	Reverse primer
Arg1	5'-GAGCACATGCAGCAGCAGCAG-3'	5'-TTTCCTCAGTGCTGCAGGGCC-3'
βcatenin	5'-GTGCAATTCCTGAGCTGACA-3'	5'-CTTAAAGATGGCCAGCAAGC-3'
Cd206	5'-TGTGGTGAGCTGAAAGGTGA-3'	5'-CAGGTGTGGGCTCAGGTAGT-3'
Claudin7	5'-CTGGTGTTGGGCTTCTTAGC-3'	5'-CAGAAGGACCAGAGCAGACC-3'
Gapdh	5'-CCTCGTCCCGTAGACAAAATG-3'	5'-TCTCACTTTGCCACTGCAA-3'
Ifnγ	5'-TCAAGTGGCATAGATGTGGAAGAA-3'	5'-TGGCTCTGCAGGATTTTCATG-3'
Il1b	5'-TCAGGCAGGCAGTATCACTCA-3'	5'-GGAAGGTCCACGGGAAAGAC-3'
Il6	5'-CTGCAAGAGACTTCCATCCAGTT-3'	5'-AAGTAGGAAGCCGTGGTT-3'
Il10	5'-CTGGGTGAGAAGTGAAGACC'-3'	5'-CATGGCCTTGTAGACACCTTG-3'
Il12b	5'-GGACTACCCTGACTCCTGGAG-3'	5'-GATGTCTTCTCTCTACGAGGAAC-3'
Il17a	5'-CCCCATGGGATTACAACATCAC-3'	5'-TTTCCTCAGTGCTGCAGGGCC-3'
Il23	5'-CCAGCAGCTCTCTCGGAATCT-3'	5'-GGGTCACAACCATCTTCACAC-3'
Muc2	5'-ACATCACCTGTCCCGACTTC-3'	5'-GAGCAAGGGACTCTGTCTG-3'
Muc3	5'-TGTTCAGCTTTACTGTGTTTCAA-3'	5'-TTGCATGTCTCCTCAGGATT-3'
Muc13	5'-TATTACTACAACTCGTCTCT-3'	5'-CTCTTGATAGCCCACAGAGT-3'
Oclaudin	5'-CCTCCAATGGCAAAGTGAAT-3'	5'-CTCCCCACCTGTCGTGTAGT-3'
Reg3g	5'-CCTTCCTCTTCCTCAGGCAAT-3'	5'-TATTCTCTCTCTCCACTTCAGAAATCCT-3'
Relmα	5'-CCCTTCTCATCTGCATCTCC-3'	5'-CAGTAGCAGTCATCCCAGCA-3'
Saa1	5'-CATTTGTTCACGAGGCTTTCC-3'	5'-GTTTTTCCAGTTAGCTTCCTTCATGT-3'
Saa2	5'-TGTGTATCCCACAAGGTTTCAGA-3'	5'-TTATTACCCTCTCCTCCTCAAGCA-3'
Saa3	5'-GCAACTACTGGGTTGAGATA-3'	5'-GAGCATGGGATTACAACATCC-3'
Tnfa	5'-TCCAGGCGGTGCCTATGT-3'	5'-CACCCCGAAGTTCAGTAGACAGA-3'
